# Improving Agricultural Traits While Maintaining High Resistant Starch Content in Rice

**DOI:** 10.1186/s12284-022-00573-5

**Published:** 2022-06-04

**Authors:** Satoko Miura, Maiko Narita, Naoko Crofts, Yuki Itoh, Yuko Hosaka, Naoko F. Oitome, Misato Abe, Rika Takahashi, Naoko Fujita

**Affiliations:** Department of Biological Production, Akita Prefectural University, Akita, 010-0195 Japan

**Keywords:** Amylopectin, Amylose, Backcrossing, Endosperm starch, Resistant starch, Rice (*Oryza sativa*), Starch branching enzyme

## Abstract

**Background:**

Resistant starch (RS) is beneficial for human health. Loss of starch branching enzyme IIb (BEIIb) increases the proportion of amylopectin long chains, which greatly elevates the RS content. Although high RS content cereals are desired, an increase in RS content is often accompanied by a decrease in seed weight. To further increase the RS content, genes encoding active-type starch synthase (SS) IIa, which elongates amylopectin branches, and high expression-type granule-bound SSI (GBSSI), which synthesizes amylose, were introduced into the *be2b* mutant rice. This attempt increased the RS content, but further improvement of agricultural traits was required because of a mixture of *indica* and *japonica* rice phonotype, such as different grain sizes, flowering times, and seed shattering traits. In the present study, the high RS lines were backcrossed with an elite rice cultivar, and the starch properties of the resultant high-yielding RS lines were analyzed.

**Results:**

The seed weight of high RS lines was greatly improved after backcrossing, increasing up to 190% compared with the seed weight before backcrossing. Amylopectin structure, gelatinization temperature, and RS content of high RS lines showed almost no change after backcrossing. High RS lines contained longer amylopectin branch chains than the wild type, and lines with active-type SSIIa contained a higher proportion of long amylopectin chains compared with the lines with less active-SSIIa, and thus showed higher gelatinization temperature. Although the RS content of rice varied with the cooking method, those of high RS lines remained high after backcrossing. The RS contents of cooked rice of high RS lines were high (27–35%), whereas that of the elite parental rice was considerably low (< 0.7%). The RS contents of lines with active-type SSIIa and high-level GBSSI expression in *be2b* or *be2b ss3a* background were higher than those of lines with less-active SSIIa.

**Conclusions:**

The present study revealed that backcrossing high RS rice lines with elite rice cultivars could increase the seed weight, without compromising the RS content. It is likely that backcrossing introduced loci enhancing seed length and width as well as loci promoting early flowering for ensuring an optimum temperature during RS biosynthesis.

**Supplementary Information:**

The online version contains supplementary material available at 10.1186/s12284-022-00573-5.

## Background

Starch stored in seeds or tubers is the major source of carbohydrates required for seed germination, and is an important source of energy in the human diet. Starch can be classified into three categories, depending on the rate of degradation by amylases and amyloglucosidases: rapidly digestible starch, which is digested within 20 min; slowly digestible starch, which is digested in 20–120 min; and resistant starch (RS), which is recalcitrant to degradation by amylases and amyloglucosidases, and is not absorbed in the small intestine (Englyst et al. 1992). RS is further categorized into five types, depending on its physical characteristics: RS1, starch physically protected by the rigid cell wall; RS2, starch possessing B-type X-ray crystallinity; RS3, retrograded and recrystallized starch formed from gelatinized starch upon cooling; RS4, chemically modified starch; and RS5, amylose–lipid complex (Englyst et al. 1992; Nugent 2005; Hasjim et al. 2010).

Currently, RS is receiving considerable attention, as it is beneficial for human health. Starch rich in RS releases glucose not only in small amounts but also at a slow rate, thus preventing sudden postprandial glucose responses (Raben et al. 1994). Therefore, intake of RS likely prevents lifestyle-related diseases and helps manage diabetes (Wilcox 2005; Wang et al. 2019). Since RS reaches the colon as polyglucans, it functions as fiber in the large intestine. RS serves as a prebiotic, and is fermented by microbiota, secreting short fatty acids such as butyrate, propionate, and acetate (Amini et al. 2016; Lockyer and Nugent 2017). These fatty acids prevent colorectal cancer by suppressing proliferation and inducing apoptosis of cancer cells through the inhibition of histone deacetylase (Hinnebusch et al. 2002; Fung et al. 2012). Furthermore, short chain fatty acids in colon also play pivotal roles in preventing food allergies by activating signaling cascades and promoting the differentiation of regulatory T and B cells, which alleviate hyperactive immune responses (Roduit et al. 2019; Luu et al. 2020). Therefore, breeding of cereals rich in RS is desired.

Starch is composed of two polymers, amylopectin and amylose. Amylopectin, the major component of starch, is a highly branched molecule with a crystalline structure, whereas amylose is essentially a linear molecule (Hizukuri 1986; Takeda et al. 1987; Nakamura and Kainuma 2021). Amylopectin is synthesized by the coordinated action of multiple starch biosynthetic enzymes: starch synthases (SSs), which elongate α-1,4 linked glucans using ADP-glucose as a substrate; starch branching enzymes (BEs), which generate α-1,6 linked branches; and debranching enzymes (DBEs), which trim off inappropriate branches (Nakamura 2002; Fujita 2014). Each class of starch biosynthetic enzymes consists of multiple isozymes with different spatiotemporal expression patterns and preferred glucan primer and end-product structures (Yamanouchi and Nakamura 1992; Ohdan et al. 2005; Crofts et al. 2017; Sawada et al. 2018). Exhaustive analyses of *japonica* rice mutants lacking starch biosynthetic enzymes have revealed the process of amylopectin biosynthesis in the rice endosperm. SSIIIa synthesizes long backbone glucans with a degree of polymerization (DP) > 30 (Fujita et al. 2007), and BEI generates long branches (Satoh et al. 2003). BEIIb generates short branches with DP 6–7 (Nishi et al. 2001), and SSI elongates these short branches to DP 8–12 (Fujita et al. 2006). These branches are further elongated to DP 12–24 by SSIIa in typical *indica* rice, which carries active-type SSIIa, but not in *japonica* rice with less-active SSIIa (Umemoto et al. 2002; Nakamura et al. 2005). Excess branches are trimmed off by isoamylase 1 (ISA1) (Nakamura et al. 1997; Nakamura 2002). Some of the starch biosynthetic enzymes form protein complexes (Crofts et al. 2015), resulting in mutually increased activity (Nakamura et al. 2012). In contrast to amylopectin, amylose is synthesized solely by granule-bound starch synthase I (GBSSI). Typical *indica* rice cultivars express GBSSI to high levels, resulting in high amylose content; however, typical *japonica* rice expresses low levels of GBSSI, resulting in low amylose content (Sano 1984; Wang et al. 1995; Isshiki et al. 1998). A balance in the activities of different starch biosynthetic enzymes is important for maintaining the amylopectin structure and amylose content, which influence the physicochemical properties of starch, ultimately determining starch application and digestibility (Tsuiki et al. 2016).

Absence of BEIIb in *japonica* rice mutants drastically increases the RS content (Tsuiki et al. 2016). The RS content of cooked rice grains of wild-type (WT) *japonica* rice is approximately 1% (Tsuiki et al. 2016; Miura et al. 2021), whereas those of *be2b*, *ss3a be2b*, *ss1*^*L*^* be2b*, and *be1 be2b* are 21–28% (Tsuiki et al. 2016; Itoh et al. 2017; Miura et al. 2021), 17% (Tsuiki et al. 2016), 34% (Tsuiki et al. 2016), and 76% (Miura et al. 2021), respectively. The RS found in *be2b* mutant lines is categorized as RS2 since all of these lines display B-type crystallinity because of the high proportion of long amylopectin branches (Nishi et al. 2001; Wei et al. 2010; Abe et al. 2013, 2014; Asai et al. 2014; Miura et al. 2021). After cooking, the RS in these lines is categorized as R3 (Englyst et al. 1992). In addition, typical *indica* rice cultivars, with high-level GBSSI expression, exhibit approximately twofold higher RS content (approximately 3%) than typical *japonica* rice cultivars, although the effect of high-level GBSSI expression on RS content was minor compared with that of BEIIb loss (Tsuiki et al. 2016; Zhou et al. 2016).

Absence of BEIIb decreases the number, but increases the length, of amylopectin branches, thus making starch retrogradation easier and degradation more difficult. Residual glucans, obtained after the treatment of starch with digestive enzymes, are enriched with long amylopectin branches (DP > 21) and partially digested amylose, although the residual glucan structure can vary, depending on the processing method, initial amylopectin structure, and amylose content (Miura et al. 2021). Increase in the RS content of *japonica be2b* mutant rice is often accompanied by a reduction in seed weight. This is because strong endosperm-specific expression of *BEIIb* results in the production of amylopectin branches and non-reducing glucan ends, which serve as substrates for SSI and enhance the mutual activity of enzymes to synthesize amylopectin (Nakamura et al. 2014). While the absence of BEIIb leads to suboptimal amylopectin biosynthesis, it creates favorable conditions for amylose synthesis because unused ADP-glucose can be used as a substrate by GBSSI for amylose biosynthesis; this reaction has a higher Michaelis–Menten constant than reactions catalyzed by other SS isozymes (Clarke et al. 1999). Therefore, increasing the amylopectin branch length and amylose content is predicted to elevate the RS content.

To further increase the RS content, the japonica *be2b* mutant (*ss2a*^*L*^* gbss1*^*L*^* be2b*) was crossed with indica rice (*SS2a GBSS1 BE2b*) to generate #1203B (*ss2a*^*L*^* GBSS1 be2b*) and #1203C (*SS2a GBSS1 be2b*) lines with active-type SSIIa and/or high-level GBSSI protein (Itoh et al. 2017). The RS contents of #1203B (*ss2a*^*L*^* GBSS1 be2b*) and #1203C (*SS2a GBSS1 be2b*) were 23–26% and 20–25%, respectively, which were slightly higher than that of the parental *japonica be2b* mutant (22%; *ss2a*^*L*^* gbss1*^*L*^* be2b*). Although the seed weights of #1203B and #1203C were higher than that of the parental *japonica be2b* mutant (56% of the *japonica* WT rice), it reached only approximately 80% of the seed weight of typical WT *japonica* rice (*ss2a*^*L*^* gbss1*^*L*^* BE2b*) (Itoh et al. 2017). These findings highlighted the potential for further improvement in RS content. However, how the introduction of gene(s) encoding active-type SSIIa (*SS2a* allele) and/or high-level GBSSI protein (*GBSS1* allele) affected the RS content remained elusive because other combinations of SS isozyme genes, such as *SS2a* *gbss1*^*L*^ *be2b*, had not been isolated at the time Itoh et al. (2017) was published. Additionally, *SS2a ss3a gbss1*^*L*^* be2b*, *ss2a*^*L*^* ss3a GBSS1 be2b*, and *SS2a ss3a GBSS1 be2b* rice lines with amylose contents higher than those of *ss2a*^*L*^* gbss1*^*L*^* be2b* had not been isolated either. Furthermore, the genetic backgrounds of #1203B and #1203C were a mixture of *indica* and *japonica* rice, resulting in a mixed phonotype, such as different grain sizes and colors, flowering times, and seed shattering traits, which made it difficult to isolate the effects of specific genes on RS content (Itoh et al. 2017).

In the present study, backcrossing of RS-rich rice lines, *be2b* and *ss3a be2b* mutant lines harboring or lacking active-type *SSIIa* and/or high-level *GBSSI* expression, was performed to accurately evaluate the effects of different combinations of starch biosynthetic genes on RS content in the same genetic background. In addition, we investigated whether and how backcrossing with the high-yielding elite rice cultivar, Akita 63, affected the agricultural traits and RS content of high RS lines. Successful candidates exhibited an increase in seed weight to levels similar to or greater than those of typical WT *japonica* rice cultivars, and the physicochemical properties and digestibility of starch in these lines were analyzed.

## Results

### Presence and Absence of SS and BE

**Fig. 1:**
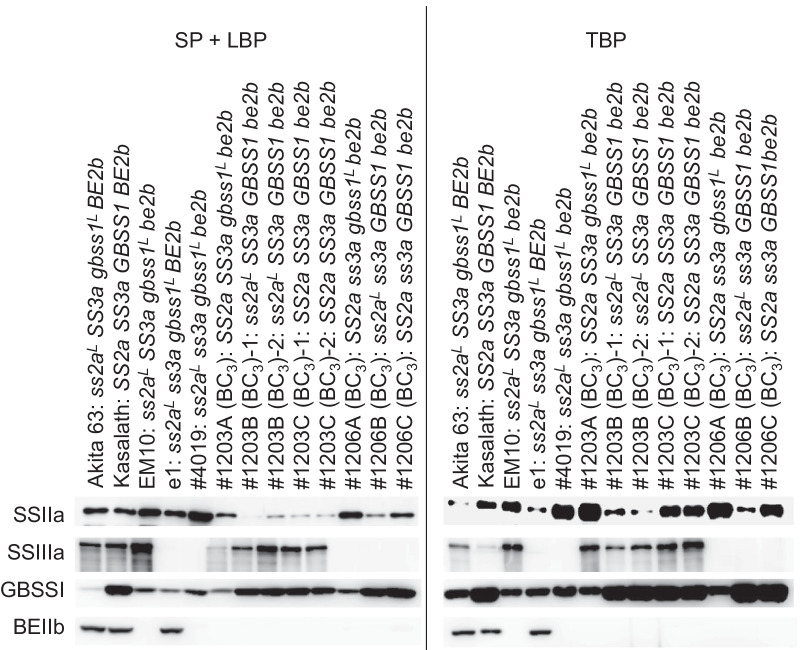
Western blotting analysis of starch biosynthetic enzymes, SSIIa, SSIIIa, GBSSI, and BEIIb, using mature seed extract. **a** Soluble protein and proteins loosely bound to the starch granule (SP + LBP). **b** Proteins tightly bound to the starch granule (TBP)

To generate high-yielding RS-rich rice lines, *be2b* and *ss3a be2b* mutant lines harboring or lacking active-type *SSIIa* and/or high-level *GBSSI* expression were backcrossed with the high-yielding elite rice cultivar Akita 63 (see pedigree in Additional file 1: Fig. S1). Homozygous plants were isolated from the backcross progeny; genotypes of all lines used in this study are summarized in Table 1. The backcrossed lines *SS2a SS3a gbss1*^*L*^* be2b*, *ss2a*^*L*^* SS3a GBSS1 be2b*, and *SS2a SS3a GBSS1 be2b* lines were designated as #1203A (BC_3_), #1203B (BC_3_), and #1203C (BC_3_), respectively. Two lines of #1203B (BC_3_) were designated as #1203B (BC_3_)-1 and #1203B (BC_3_)-2, and two lines of #1203C (BC_3_) were designated as #1203C (BC_3_)-1 and #1203C (BC_3_)-2. The backcrossed lines *SS2a ss3a gbss1*^*L*^* be2b*, *ss2a*^*L*^* ss3a GBSS1 be2b*, and *SS2a ss3a GBSS1 be2b* were designated as #1206A (BC_3_), #1206B (BC_3_), and #1206C (BC_3_), respectively. The absence of SSIIIa and/or BEIIb was verified by western blotting analysis of the extract containing soluble protein and proteins loosely bound to the starch granule (SP + LBP) (Fig. 1). Western blotting using anti-SSIIIa antibody showed that SSIIIa was absent in e1, #4019, and all three #1206 (BC_3_) lines (Fig. 1). Similarly, western blotting using anti-BEIIb antibody confirmed that BEIIb was absent in EM10, #4019, and all #1203 (BC_3_) and #1206 (BC_3_) lines (Fig. 1). GBSSI and active-type SSIIa are known to be tightly associated with starch (Bao et al. 2009; Itoh et al. 2017; Crofts et al. 2019). Western blotting of proteins tightly bound to starch (TBP) using anti-SSIIa antibody revealed greater abundance of SSIIa in rice lines with the *SS2a* genotype (i.e., containing active-type SSIIa), such as Kasalath, #1203A (BC_3_), #1203C (BC_3_), #1206A (BC_3_), and #1206C (BC_3_), than in lines with the *ss2a*^*L*^ genotype such as Akita 63, e1, #1203B (BC_3_), and #1206B (BC_3_). Absence of BEIIb also increased the amount of SSIIa protein in the TBP fraction of EM10 and #4019, which is consistent with previous studies (Asai et al. 2014; Itoh et al. 2017). GBSSI was enriched in the TBP fraction, and lines with the *GBSS1* genotype, such as Kasalath, #1203B (BC_3_), #1203C (BC_3_), #1206B (BC_3_), and #1206C (BC_3_), contained higher levels of the GBSSI protein than lines with the *gbss1*^*L*^ genotype, such as Akita 63, EM10, e1, #4019, #1203A (BC_3_), and #1206A (BC_3_). These results show that all lines used in this study possess the correct genotype and exhibit the expected patterns of SSIIa, SSIIIa, GBSSI, and BEIIb protein abundance.

**Table 1:** Genotypes of rice lines used in this study, and their grain weight-related data

Line	Genotype	Grain weight^a^		Rate of increase (%)^b^
		Mean ± SE	%	
Kinmaze	*ss2a*^*L*^* SS3a gbss1*^*L*^* BE2b*	19.0 ± 0.3e	100	–
Nipponbare	*ss2a*^*L*^* SS3a gbss1*^*L*^* BE2b*	20.0 ± 0.3de	105	–
Akita 63	*ss2a*^*L*^* SS3a gbss1*^*L*^* BE2b*	28.4 ± 0.3a	149	–
Kasalath	*SS2a SS3a GBSS1 BE2b*	15.4 ± 0.2f	81	–
EM10 (BC_3_)	*ss2a*^*L*^* SS3a gbss1*^*L*^* be2b*	19.4 ± 0.6e	102	181
#4019 (BC_3_)	*ss2a*^*L*^* ss3a gbss1*^*L*^* be2b*	21.3 ± 0.3bcde	112	134
#1203A (BC_3_)	*SS2a SS3a gbss1*^*L*^* be2b*	12.7 ± 0.3g	67	135
#1203B (BC_3_)-1	*ss2a*^*L*^* SS3a GBSS1 be2b*	28.1 ± 0.4a	148	184
#1203B (BC_3_)-2	*ss2a*^*L*^* SS3a GBSS1 be2b*	29.0 ± 0.8a	153	190
#1203C (BC_3_)-1	*SS2a SS3a GBSS1 be2b*	22.2 ± 0.6bcd	117	144
#1203C (BC_3_)-2	*SS2a SS3a GBSS1 be2b*	23.6 ± 0.4b	124	153
#1206A (BC_3_)	*SS2a ss3a gbss1*^*L*^* be2b*	19.1 ± 0.4e	101	145
#1206B (BC_3_)	*ss2a*^*L*^* ss3a GBSS1 be2b*	22.9 ± 0.5bc	121	132
#1206C (BC_3_)	*SS2a ss3a GBSS1 be2b*	20.7 ± 0.3cde	109	145

^a^The weight (mg) of one dehulled mature grain of each rice line is presented as mean ± standard error (SE; *n* = 20) as well as a percentage of the seed weight of the wild-type (WT) cultivar, Kinmaze. Increase in seed weight before and after backcrossing was calculated. Different lowercase letters indicate significant differences among the different rice genotypes (*P* < 0.05; Tukey–Kramer method)

^b^Rate of increase in seed weight after backcrossing relative to that before backcrossing relative to the values in Table S1. Average seed weight was used where multiple lines with the same genotype were available

### Seed Morphology and Seed Weight

**Fig. 2:**
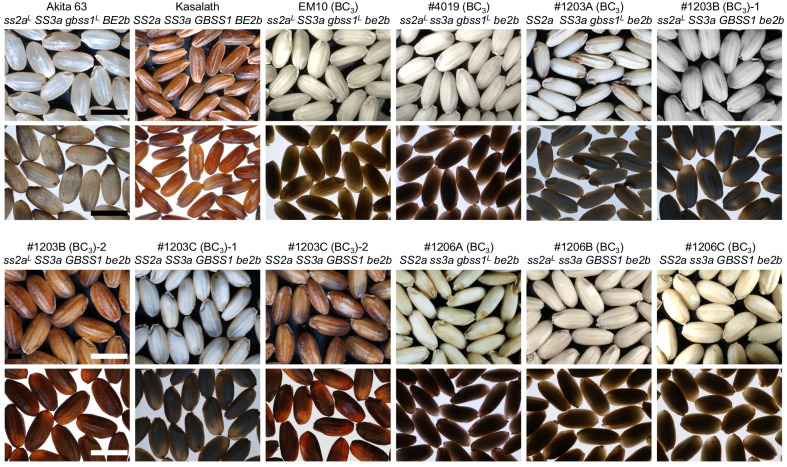
Morphology of dehulled mature seeds. Photos of seeds of different rice lines captured using light from above (upper panels) and below (lower panels). Scale bars: 5 mm

The seeds of all rice lines lacking BEIIb were opaque, whereas those of the WT, Akita 63, and Kasalath were translucent (Fig. 2). Seeds of #1203B (BC_3_)-2 and #1203C (BC_3_)-2 showed red pericarp, a trait inherited from Kasalath. All BC_3_ lines produced large short-grains, except Kasalath, which produced long-grains, and #1203A (BC3), whose grains were smaller than those of other lines and sometimes flat (Fig. 2). The seed weight of Akita 63 was high (28 mg per grain) and was 149% of the seed weight of the typical *japonica* rice cultivar, Kinmaze (19 mg per grain) (Table 1). The seed weights of all RS-rich lines after backcrossing were greatly improved, with an approximately 132–190% increase (EM10, 181%; #4019, 134%, #1203A, 135%, #1203B, up to 190%; #1203C, up to 153%; #1206A, 145%; #1206B, 132%; and #1206C, 145%; Table 1), compared with those of RS-rich lines before backcrossing (Additional file 2: Table S1). The rate of increase in seed weight can be influenced by the initial seed weight; if the initial seed weight is high, the rate of increase may not be large. The seed weights of almost all lines, except for #1203A (BC_3_), after backcrossing were similar to or heavier than that of the typical *japonica* rice, Kinmaze (Table 1). The #1203B (BC_3_) lines produced the largest seeds among all backcrossed lines, and weighed 28–29 mg per seed on average, which was similar to the seed weight of Akita 63. Seed weight of #1203A was 12.7 mg per seed on average, which was the lowest among all backcrossed lines, although its seed weight was increased by 135% after backcrossing compared with that before backcrossing. These results indicate that backcrossing successfully increased the seed weight of high RS lines, which was probably caused by the inheritance of loci responsible not only for large seed size but also for early flowering, which would ensure optimum temperature during starch synthesis. The backcrossed lines flowered in the beginning of August, approximately 10–30 days earlier than the flowering time of lines before backcrossing. Early flowering was accompanied by an increase in cumulative temperature at 5–30 days after flowering (DAF), when endosperm starch biosynthesis is at its peak. Cumulative temperature of after backcrossing was 680–693 °C, but that of before backcrossing was 452–640 °C (Additional file 2: Table S2).

### Amylose Content and Ratio of Short-to-Long Amylopectin Chains

**Fig. 3:**
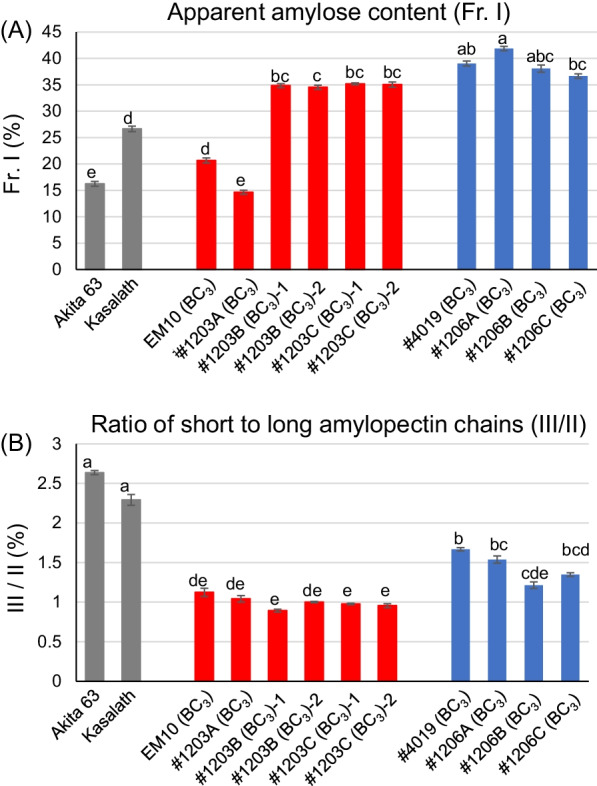
Measurement of apparent amylose content by gel filtration chromatography using debranched starch. Fraction I contains amylose and extra-long chains of amylopectin; fraction II contains long chains of amylopectin; and fraction III contains short chains of amylopectin. **a** Apparent amylose content. **b** Ratio of short to long amylopectin chains, calculated by dividing fraction III value by fraction II value. Different lowercase letters indicate significant differences (*P* < 0.05; Tukey–Kramer method)

The expression level of *GBSSI* is influenced by the temperature during seed development. When the temperature during the seed development is high, RNA splicing of *GBSSI* is compromised, resulting in reduced levels of mature *GBSSI* mRNA and consequently GBSSI protein, which decreases the amylose content (Hirano and Sano 1998; Larkin and Park 1999; Zhang et al. 2014; Kato et al. 2019). Since high amylose content is known to contribute to the elevation in RS content (Tsuiki et al. 2016; Zhou et al. 2016; Chen et al. 2017), the apparent amylose contents of high RS lines after backcrossing were analyzed by gel filtration chromatography using debranched starch (Fig. 3, Additional file 2: Table S3), and compared with those from before backcrossing as well as with those of rice lines harboring different combinations of starch biosynthetic enzymes (Additional file 2: Table S4). Elution profiles of different rice lines are shown in Additional file 1: Fig. S2, where fractions I, II, and III mainly represent amylose, amylopectin long chains, and amylopectin short chains, respectively.

Apparent amylose contents of *ss3a be2b* lines, such as #4019 and #1206, were higher than those of *be2b* lines, such as EM10 and #1203, both before and after backcrossing (Fig. 3, Additional file 2: Tables S3, S4). The ratio of short to long amylopectin chains in *be2b* lines, such as EM10 and #1203, were lower (0.8–1.2) than that in *ss3a be2b* lines, such as #4019 and #1206 (1.1–1.5), regardless of backcrossing (Additional file 2: Tables S3, S4). These results indicate that #1203 lines contain more long amylopectin chains than #1206 lines. Among the *be2b* lines, amylose contents of lines with the *GBSS1* genotype, such as #1203B and #1203C, were higher (35–39%) than those of the *gbss1*^*L*^ lines, such as EM10 (20–27%) and #1203A (15–16%), and the apparent amylose content of #1203A was lower than that of EM10, regardless of backcrossing (Fig. 3, Additional file 2: Tables S3, S4). Apparent amylose contents of all *ss3a be2b* lines were high, and no significant differences were observed among lines with *GBSS1* or *gbss1*^*L*^ alleles (Fig. 3, Additional file 2: Tables S3, S4). This may be because the absence of SSIIIa elevates AGPase activity, and the increased ADP-glucose levels enhance amylose biosynthesis even under the low expression levels of *GBSSI* (Crofts et al. 2021a). Moreover, a high expression level of *GBSSI* may not be able to further increase the apparent amylose content because of a limitation in the amount of ADP-glucose.

The apparent amylose content of almost all the lines decreased slightly after backcrossing (Additional file 2: Tables S3, S4), most likely because of the high temperature during seed development (Additional file 2: Table S2).

### Amylopectin Branch Structure

**Fig. 4:**
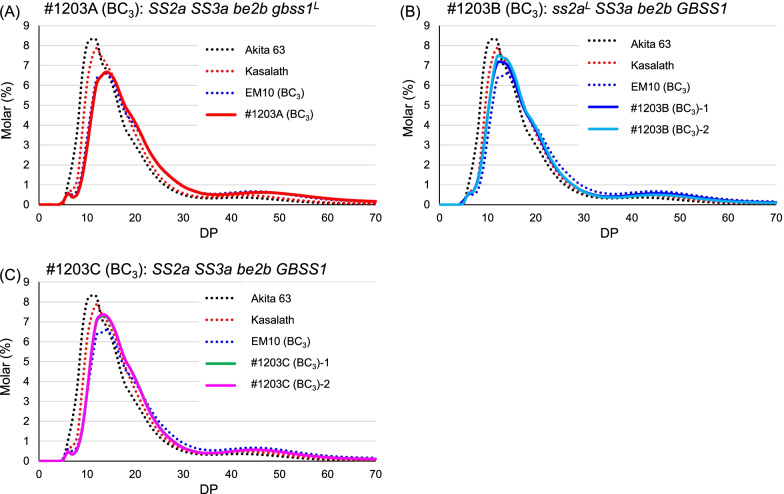
Comparison of chain length distribution patterns of endosperm amylopectin in #1203 (BC_3_) lines with those in Akita 63, Kasalath, and EM10 (BC_3_). **a**–**c** Chain length distribution patterns of #1203A (BC_3_) (**a**), #1203B (BC_3_) (**b**), and #1203C (BC_3_) (**c**). Typical patterns obtained in one of at least three replicates are shown

To reveal the detailed amylopectin branch structure, starch was debranched and analyzed by capillary electrophoresis. Compared with the chain length distribution pattern of Akita 63, the peak amylopectin chain length of EM10 (BC_3_) and #1203 (BC_3_) lines, which lack BEIIb, increased by three glucose residues from DP 11 to DP 14 (Fig. 4), and the height of these peaks was considerably lower than that of Akita 63. This indicates that the loss of BEIIb reduced the number of short chains and increased the number of long chains in EM10 (BC_3_) and #1203 (BC_3_) lines compared with their distribution in Akita 63 (Fig. 4). The chain length distribution patterns of #1203 (BC_3_) lines were essentially the same as those before backcrossing, and only a minor increase was seen (DP < 20) (Additional file 1: Fig. S3A).

To determine differences between the chain length distribution patterns of #1203 (BC_3_) lines and Akita 63, subtraction curves were generated by subtracting the values of Akita 63 from those of #1203 (BC_3_) lines (Additional file 1: Fig. S4). The number of short chains (DP < 13) was considerably reduced, while that of long chains (DP > 15) was increased in #1203 (BC_3_) lines compared with their numbers in Akita 63 (Additional file 1: Fig. S4). The chain length distribution pattern of Kasalath shifted toward the larger side by two glucose residues compared with Akita 63 (Fig. 4), and the subtraction curve generated by subtracting the value of Akita 63 from that of Kasalath showed that the number of short chains in Akita 63 was greater than that in Kasalath. This is in agreement with a previous study (Nakamura et al. 2002, 2005), since active-type SSIIa functions to elongate the amylopectin branches from DP 6–12 to DP 12–24. The rate of reduction of short chains relative to Akita 63 was greater in #1203A (BC_3_) and #1203C (BC_3_) than in #1203B (BC_3_), indicating that active-type SSIIa uses short chains and generates longer chains. To determine the effect of active SSIIa on amylopectin structure in the *be2b* background, subtraction curves were generated as “#1203A (BC_3_)—EM10 BC_3_” and “#1203C (BC_3_)—#1203B (BC_3_)” and compared with “Kasalath—Akita 63” (Additional file 1: Fig. S5A). Lines with active-type SSIIa contained less short amylopectin branches (DP ≤ 12) and more long amylopectin branches (13 < DP < 30), indicating that active SSIIa synthesizes long amylopectin branches (13 < DP < 30) from short amylopectin chains (DP ≤ 12). However, the absolute values of molar % were much smaller in the absence of BEIIb compared with “Kasalath – Akita 63” (Additional file 1: Fig. S5A). This was perhaps caused by the BEIIb loss-induced absence of short branches and by the small number of glucans that can serve as a primer for SSIIa.

**Fig. 5:**
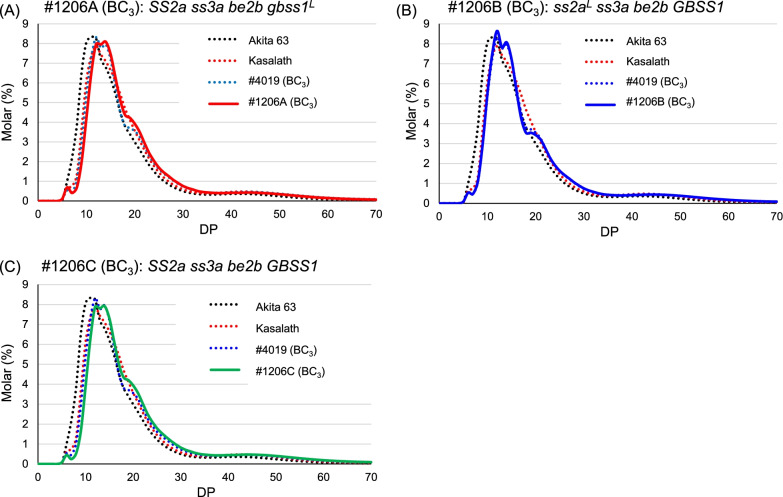
Comparison of chain length distribution patterns of endosperm amylopectin in #1206 (BC_3_) lines with those in Akita 63, Kasalath, and #4019 (BC_3_). **a–c** Chain length distribution patterns of #1206A (BC_3_) (**a**), #1206B (BC_3_) (**b**), and #1206C (BC_3_) (**c**). Typical patterns obtained in one of at least three replicates are shown

The chain length distribution patterns of #4019 (BC_3_) and #1206 (BC_3_) lines, which lack SSIIIa and BEIIb, also shifted toward the larger side by approximately two glucose residues, from DP 11 to DP 12 or 14, compared with that of Akita 63 (Fig. 5). In contrast to #1203 (BC_3_), lines #4019 (BC_3_) and #1206 (BC_3_) showed higher peaks at DP 14 and a characteristic shoulder at DP 19 (Fig. 5). The chain length distribution patterns of #1206 (BC_3_) lines were essentially the same as those before backcrossing, and only a minor increase was detected (DP < 20) (Additional file 1: Fig. S3B).

To determine differences between the chain length distribution patterns of #1206 (BC_3_) lines and Akita 63, subtraction curves were generated by subtracting the values of Akita 63 from those of #1206 (BC_3_) lines (Additional file 1: Fig. S6). The number of short chains (DP ≤ 12) was greatly reduced, and that of long chains (DP > 15) were increased in #1206 (BC_3_) lines compared with their numbers in Akita 63 (Additional file 1: Fig. S6). The rate of reduction of short chains relative to Akita 63 was greater in #1206A (BC_3_) and #1206C (BC_3_) lines than in #1206B (BC_3_), indicating that active-type SSIIa uses short chains and generates longer chains. To determine the effect of active SSIIa on amylopectin structure in the *ss3a be2b* background, subtraction curves were generated as “#1206A (BC_3_)—#4019 BC_3_” and “#1206C (BC_3_)—#1206B (BC_3_)” and compared with “Kasalath—Akita 63” (Additional file 1: Fig. S5B). Lines with active SSIIa contained less short amylopectin branches (DP ≤ 12) and more long amylopectin branches (15 < DP < 30), although the effects of active SSIIa in these lines were milder than that in Kasalath but stronger than those in #1203 (BC_3_) lines (Additional file 1: Fig. S5).

To compare the effect of *GBSS1* or *gbss1*^*L*^ alleles in the absence of only BEIIb or both BEIIb and SSIIIa, subtraction curves were generated (Additional file 1: Fig. S7). In lines carrying *GBSS1* but lacking *BEIIb*, “#1203B (BC_3_)—EM10 (BC3)” and “#1203C (BC_3_)—#1203A (BC_3_)” showed more amylopectin chains with 8 < DP < 20 and less amylopectin chains with DP > 20 (Additional file 1: Fig. S7A). By contrast, the absolute values of “#1206B (BC_3_)—#4019 (BC_3_)” and “#1206C (BC_3_)—#1206A (BC_3_)” were small, suggesting that GBSSI protein levels did not affect amylopectin structure in the absence of SSIIIa and BEIIb (Additional file 1: Fig. S7B).

### Thermal Properties of Total Starch

Difference in the amylopectin branch length is strongly correlated with the gelatinization temperature of starch and ease of its retrogradation, both of which are affected by the temperature during seed development (Nakamura et al. 2005; Umemoto et al. 2008; Chun et al. 2015). Since retrograded starch also serves as RS (Englyst et al. 1992), the thermal properties of purified starch were analyzed by differential scanning calorimetry, and compared among lines harboring different combinations of starch biosynthetic genes both before and after backcrossing (Table 2, Additional file 2: Table S5, Additional file 1: Fig. S8). Loss of BEIIb in EM10 (BC_3_) and presence of *SS2a* allele in Kasalath result in higher gelatinization temperature than Akita 63. The gelatinization temperature of EM10 (BC_3_) and #1203 (BC_3_) lines was approximately 7 °C higher than that of #4019 (BC_3_) and #1206 (BC_3_) lines (Table 2, Additional file 1: Fig. S8), most likely because EM10 (BC_3_) and #1203 (BC_3_) lines contained less short amylopectin chains (DP ≤ 12) and more long amylopectin chains (DP > 13) compared with #4019 (BC_3_) and #1206 (BC_3_) lines (Figs. 4, 5). The gelatinization temperature of #1203A (BC_3_) was 3 °C higher than that of EM10 (BC_3_) and other #1203 (BC_3_) lines (Table 2), while the gelatinization temperature of #1206B (BC_3_) was 3 °C lower than that of #4019 (BC_3_) and other #1206 (BC_3_) lines (Table 2). The gelatinization temperature of starch in all the lines increased by 2–4 °C after backcrossing, possibly because of the higher ripening temperature accompanied by earlier flowering time, although the gelatinization temperature of Kasalath was approximately 1 °C higher or lower, depending on the growth year (Table 2, Additional file 2: Table S5, Additional file 1: Fig. S8). These results were obtained possibly because the amount of amylopectin chains with DP > 13 was higher in #1203A (BC_3_) than in #1203B (BC_3_) or #1203C (BC_3_) (Additional file 1: Figs. S4, S5, S7), and the amount of amylopectin chains with DP > 13 was less in #1206B (BC_3_) than in #1206A (BC_3_) or #1206C (BC_3_) (Fig. 5, Additional file 1: Fig. S5). These findings are consistent with previous studies, in which an increase in the abundance of amylopectin chains with 13 < DP < 25 resulted in an increase in gelatinization temperature (Hayashi et al. 2015).

**Table 2:** Thermal properties of starch analyzed by differential scanning calorimetry

Line	Genotype	*T*_*O*_ (°C)^a^	*T*_*P*_ (°C)^a^	*T*_*C*_ (°C)^a^	*Δ*H (J/g)^a^
Akita 63	*ss2a*^*L*^* SS3a gbss1*^*L*^* BE2b*	57.7 ± 0.0cd	64.0 ± 0.0f	71.0 ± 0.1c	14.2 ± 0.3a
Kasalath	*SS2a SS3a GBSS1 BE2b*	66.2 ± 0.0ab	70.2 ± 0.0c	74.2 ± 0.4bc	14.3 ± 1.0a
EM10 (BC_3_)	*ss2a*^*L*^* SS3a gbss1*^*L*^* be2b*	64.0 ± 0.3b	72.5 ± 0.2b	80.9 ± 0.1a	12.0 ± 0.4a
#4019 (BC_3_)	*ss2a*^*L*^* ss3a gbss1*^*L*^* be2b*	56.7 ± 0.1d	66.0 ± 0.2e	80.3 ± 1.3a	10.9 ± 0.3a
#1203A (BC_3_)	*SS2a SS3a gbss1*^*L*^* be2b*	68.5 ± 0.2a	75.7 ± 0.1a	81.1 ± 0.1a	12.7 ± 0.7a
#1203B (BC_3_)-1	*ss2a*^*L*^* SS3a GBSS1 be2b*	65.9 ± 0.1ab	72.0 ± 0.1b	77.0 ± 0.0ab	11.7 ± 0.5a
#1203B (BC_3_)-2	*ss2a*^*L*^* SS3a GBSS1 be2b*	66.0 ± 0.0ab	72.6 ± 0.0b	78.0 ± 0.2ab	12.2 ± 0.7a
#1203C (BC_3_)-1	*SS2a SS3a GBSS1 be2b*	66.6 ± 0.7ab	72.6 ± 0.2b	77.6 ± 0.4ab	12.3 ± 0.7a
#1203C (BC_3_)-2	*SS2a SS3a GBSS1 be2b*	65.9 ± 0.3ab	72.6 ± 0.0b	78.3 ± 0.4ab	13.0 ± 1.6a
#1206A (BC_3_)	*SS2a ss3a gbss1*^*L*^* be2b*	60.0 ± 0.3c	68.8 ± 0.1d	74.9 ± 0.2bc	9.6 ± 1.0a
#1206B (BC_3_)	*ss2a*^*L*^* ss3a GBSS1 be2b*	58.8 ± 0.1cd	65.6 ± 0.1e	71.7 ± 0.2c	9.5 ± 0.6a
#1206C (BC_3_)	*SS2a ss3a GBSS1 be2b*	60.0 ± 0.3c	68.4 ± 0.1d	74.3 ± 0.4bc	10.0 ± 0.8a

^a^Data represent mean ± SE (*n* = 3). Different lowercase letters indicate significant differences among rice genotypes (*P* < 0.05; Tukey–Kramer method). *To*, onset temperature; *Tp*, peak temperature; *Tc*, conclusion temperature; *ΔH*, gelatinization enthalpy of starch

### RS Content

**Fig. 6:**
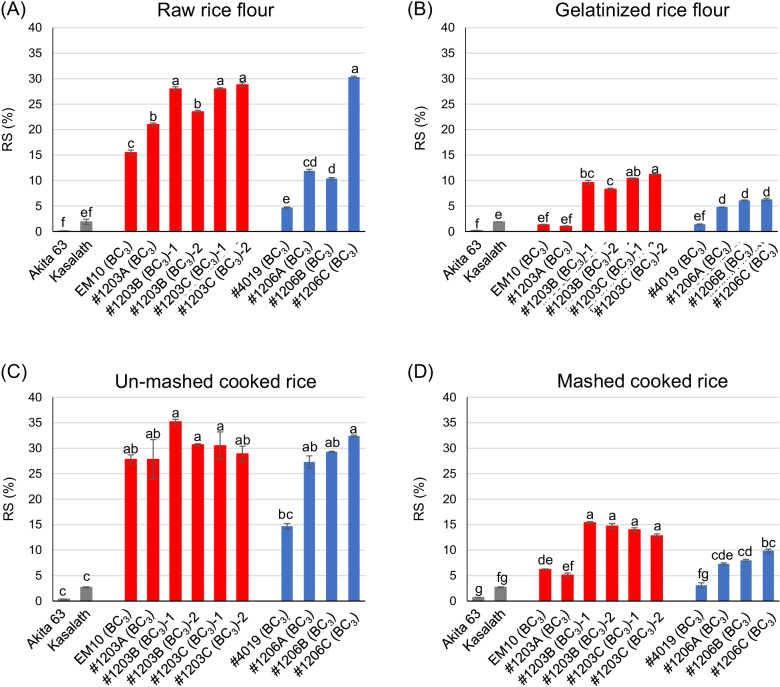
RS contents of rice processed using different methods. **a**–**d** RS contents of raw rice flour (**a**), gelatinized rice flour (**b**), un-mashed cooked rice (**c**), and mashed cooked rice (**d**). Data represent mean ± standard error (SE; *n* = 3). Different lowercase letters indicate significant differences among rice genotypes (*P* < 0.05; Tukey–Kramer method)

The RS content of rice grains varies drastically, depending on the method of cooking, which alters the accessibility of digestive enzymes to starch (Rashmi and Urooj 2003; Miura et al. 2021). Therefore, RS contents in total starch were measured using raw and gelatinized rice flour and un-mashed and mashed cooked rice. Overall, RS contents of high RS lines, such as EM10 (BC_3_), #4019 (BC_3_), #1203 (BC_3_), and #1206 (BC_3_) lines, were considerably higher (up to 290-fold) than that of Akita 63, regardless of the processing method (Fig. 6). RS contents decreased when the rice was processed (Fig. 6; Additional file 2: Table S6). RS contents of raw rice flour (5–29%) and un-mashed cooked rice (15–35%) were higher than those of gelatinized rice flour (1–11%) and mashed cooked rice (3–15%), respectively (Fig. 6, Additional file 2: Table S6), although the RS content of Akita 63 was negligible (< 0.7%) and that of Kasalath was low (< 2.7%), regardless of the processing method.

RS contents of raw rice flour samples of #1203A (BC_3_) and #1206A (BC_3_) lines, with active-type SSIIa, were higher than those of EM10 (BC_3_) and #4019 (BC_3_), with less-active type SSIIa, respectively. The RS content of the raw rice flour of #1206C (BC_3_), with high GBSSI level, was also higher than that of #1206A (BC_3_), with low GBSSI levels (Fig. 6A). These results suggest that the RS content of raw rice flour is elevated upon the increase in the contents of long amylopectin chains and amylose. When rice flour was gelatinized and cooked rice was mashed, RS contents of EM10 (BC_3_) and #1203A (BC_3_) became considerably lower than those of #1203B (BC_3_) and #1203C (BC_3_) (Fig. 6B, D). RS contents of gelatinized rice flour and mashed cooked rice samples of #4019 (BC_3_) were also lower than those of #1206 (BC_3_) lines (Fig. 6B). It can be speculated that amylose and long amylopectin branches interact to form imperfect helices during retrogradation, which prevents degradation from digestive enzymes, and serves as RS2 and RS3, when BEIIb is absent. By contrast, the number of long amylopectin chains was less in lines lacking both SSIIIa and BEIIb, allowing easier digestion, compared with lines lacking BEIIb alone. Among all of the processing methods tested, un-mashed cooked rice showed the highest RS content, regardless of the genotype, because the RS in un-mashed cooked rice exhibits aspects not only of RS1 but also of RS2 and RS3. The RS contents of un-mashed cooked rice samples of *be2b* lines, such as EM10 (BC_3_) and all #1203 (BC_3_), were high and similar (Fig. 6C), whereas the RS content of un-mashed cooked rice of #4019 (BC_3_) was lower than those of #1206 (BC_3_) lines (Fig. 6C). The RS contents of cooked un-mashed rice of EM10, #1203B, and #1203C lines before backcrossing varied from 20–26% (Itoh et al. 2017), and the levels of RS content were slightly increased by backcrossing (Fig. 6, Additional file 2: Table S6; Itoh et al. 2017). The results of the present study imply that the selection of rice cultivars for specific food applications should be based on not only their RS content but also their starch and RS structure.

## Discussion

### Improving the Seed Weight of High RS Rice Lines

The loss of BEIIb alone and in combination with other starch biosynthetic enzymes such as SSI, SSIIIa, and BEI in *japonica* rice is known to increase the RS content (Tsuiki et al. 2016; Itoh et al. 2017; Miura et al. 2021). According to our understanding, the branch length and branch frequency of amylopectin greatly affect the RS content; the longer the amylopectin branch and less frequent the branch, the higher the RS content. High amylose content also contributes to elevated RS content (Tsuiki et al. 2016; Zhou et al. 2016; Chen et al. 2017; Miura et al. 2021). A fine balance between branch generation and elongation during starch synthesis can impact seed weight, at least partly, because starch biosynthetic enzymes form multi-protein complexes to increase mutual activities synergistically for efficient starch biosynthesis (Nakamura et al. 2012, 2014; Crofts et al. 2015). Seed weight of *japonica* rice mutants lacking *BEIIb* alone is approximately half that of WT rice seeds (Additional file 2: Table S2; Nishi et al. 2001), while the loss of SSI, SSIIa, SSIIIa, or BEI in addition to that of BEIIb can alleviate the reduction in starch content, thus minimizing the reduction in seed weight (Abe et al. 2014; Asai et al. 2014; Miura et al. 2021; Ida et al. 2021).

Typical *japonica* rice cultivars exhibit low SSIIa and low GBSSI activities, resulting in shorter amylopectin branches and lower amylose content, compared with typical *indica* rice cultivars (Isshiki et al. 1998; Nakamura et al. 2005). Introduction of gene(s) encoding active-type SSIIa (*SS2a* allele) and high GBSSI proteins (*GBSS1* allele) into *japonica ss3a ss4b* or *isa1* mutant has been shown to elongate the amylopectin branches and increase amylose content and seed weight (Itoh et al. 2017; Crofts et al. 2021a, b). Therefore, introduction or deletion of specific starch biosynthetic genes can alter the starch structure and increase the seed weight.

To further elongate amylopectin branches and increase amylose content in *japonica be2b* mutant for the purpose of elevating the RS content, active-type *SS2a* allele and/or high expression-type *GBSS1* allele was introduced into the *be2b* mutant by crossing with the *indica* rice cultivar, Kasalath (Itoh et al. 2017). Although active-type SSIIa and/or high GBSSI level increased the RS content upto 4% and seed weight from 10 to 15 mg by 1.5-fold, the seed weights of the progeny were still three quarter of the typical *japonica* rice (Additional file 2: Table S1; Itoh et al. 2017). This was partly because the progeny had a mixture of *indica* and *japonica* backgrounds, as these lines were screened for specific sets of starch biosynthetic genes. For example, Kasalath grains exhibit high length-to-width ratio (Fig. 2) because expansion of seed width in Kasalath is suppressed by *qSW5* (Shomura et al. 2008). Thus, seed weight of Kasalath is only 80% of that of typical *japonica* rice (Table 1). Furthermore, heading date is fine-tuned by a combination of genes responsible for the promotion and suppression of heading in response to day length and vegetative organ size. These genes exhibit nucleotide polymorphisms between *indica* rice (Kasalath) and *japonica* rice (Nipponbare) (reviewed by Hori et al. 2016). Thus, in the current study, the heading dates of #1203 and #1206 lines before backcrossing varied considerably, ranging from mid-August to late-September. These differences in heading dates directly affected the cumulative temperature during seed development because ambient temperature rapidly declined in early September (Additional file 2: Table S2). Low temperature during seed development slows down the starch biosynthesis rate and prolongs the grain-filling period (Ahmed et al. 2008). Thus, early flowering time is beneficial at high latitude.

To overcome all of the above-mentioned factors, sequential backcrossing was conducted with the high-yielding, large seeded, and early flowering elite rice cultivar, Akita 63, which does not exhibit the grain size and yield trade-off. Enlarged grains size in Akita 63 is due to loss of function mutations in *GS3* and *qSW5* genes which determines the grain size (Makino et al. 2020). In addition, Akita 63 also shows superior nitrogen uptake capacity, and its yield is 150% of that of the commonly consumed *japonica* rice cultivar, Akitakomachi, under the same fertilizer condition (Makino et al. 2020). Thus, backcrossing with Akita 63 can lead to the development of environment friendly rice cultivars. The seed weight of all lines considerably increased after backcrossing; seeds of #1203B (BC_3_) lines were 150% heavier (28–29 mg per grain) than those of typical WT *japonica* rice (19–20 mg per grain) (Table 1, Additional file 2: Table S1). Thus, backcrossing with Akita 63 was successful in the present study. However, whether the backcrossed rice lines will maintain the high yield and RS content in subtropical or northern regions, with different climate and day length, remains to be elucidated. Elite rice cultivars for backcrossing should be carefully selected, based on their growth environment, since amylopectin structure and amylose content can be affected by the ambient temperature during seed development.

### Effects of Amylopectin Structure, Thermal Properties, and Amylose Content on RS Content

The chain length distribution patterns of high RS rice lines before and after backcrossing were essentially the same, with a minor increase in DP after backcrossing (Additional file 1: Fig. S3). The gelatinization temperature of all lines increased slightly (by 2–4 °C) after backcrossing, probably because of higher ripening temperature accompanied by the earlier flowering time (Additional file 2: Table S2). This likely enhanced the expression levels and/or activities of other starch biosynthetic enzymes, such as SSI, which increased the proportion of amylopectin chains with 8 < DP < 20 (Yamakawa et al. 2007; Kato et al. 2019).

Compared with Akita 63, EM10 (BC_3_) and #1203 (BC_3_) lines showed a considerable decline in short amylopectin chains (DP ≤ 12) and an increase in long amylopectin chains (12 < DP < 30) (Fig. 4, Additional file 1: Fig. S4). However, #4019 (BC_3_) and #1206 (BC_3_) lines contained a higher amount of short chains and fewer long chains than EM10 (BC_3_) and #1203 (BC_3_) (Fig. 5, Additional file 1: Fig. S6). The loss of SSIIIa enhances SSI activity (Fujita et al. 2007), which could explain why the number of short chains (DP ≤ 12) was higher in #4019 (BC_3_) and #1206 (BC_3_) than in EM10 (BC_3_) and #1203 (BC_3_). A decrease in the proportion of short chains in EM10 (BC_3_) and #1203 (BC_3_) lines influenced their gelatinization temperature, which was approximately 7 °C higher than that of #4019 (BC_3_) and #1206 (BC_3_) lines (Table 2, Additional file 1: Fig. S8). Differences in chain length distribution patterns also affected the RS content; RS contents gelatinized rice flour and mashed cooked rice of lines lacking BEIIb (#1203) were higher than those of lines lacking both SSIIIa and BEIIb (#1206) (Fig. 6, Additional file 2: Table S6).

High expression level of GBSSI also contributed to the increase in RS content. Comparison of apparent amylose and RS content among the *be2b* lines harboring the same *SSIIa* alleles but different *GBSSI* alleles, such as EM10 (BC_3_) vs. #1203B (BC_3_) and #1203A (BC_3_) vs. #1203C (BC_3_), showed that lines with the high-expressing *GBSS1* allele contained higher amylose and RS contents than lines possessing the low-expressing *gbss1*^*L*^ allele. The amylose contents of *ss3a be2b* lines, #4019 (BC_3_) and #1206 (BC_3_), were high (37–42%), regardless of the *GBSSI* alleles (Fig. 3, Additional file 2: Table S3). This is because the level of GBSSI protein increases in the absence of SSIIIa (Asai et al. 2014), regardless of the allele type (*GBSS1* or *gbss1*^*L*^). The loss of SSIIIa also increases AGPase activity (Asai et al. 2014; Crofts et al. 2021a). These suggest that amylose was efficiently synthesized in #4019 (BC_3_) and #1206A (BC_3_) lines, even though GBSSI proteins levels were less than that of #1206B (BC_3_) and #1206C (BC_3_) lines (Fig. 1). Nevertheless, among the *ss3a be2b* lines, those harboring the high-expressing *GBSS1* allele showed higher RS content than those possessing the low-expressing *gbss1*^*L*^ allele (Fig. 6), although the apparent amylose contents of all *ss3a be2b* lines were similar. Since the trough between fractions I and II in the gel filtration chromatography elution profiles was higher in lines harboring the *GBSS1* allele than in lines possessing the *gbss1*^*L*^ allele (Additional file 1: Fig. S2), glucans such as long amylopectin chains or short amylose may have been synthesized by GBSSI, thus contributing to the formation of RS. This is consistent with the previous study (Itoh et al. 2017), in which the high expression level of *GBSSI* likely contributed to the elongation of long amylopectin chains in #1203B and #1203C lines before backcrossing.

The RS contents of cooked un-mashed rice of EM10, #1203B, and #1203C lines before backcrossing varied from 20–26% (Itoh et al. 2017), and the levels of RS content were slightly increased by backcrossing (Fig. 6, Additional file 2: Table S6; Itoh et al. 2017). Although amylose contents decreased slightly after backcrossing, the proportion of long amylopectin chains slightly increased, thus the levels of RS content were slightly increased by backcrossing (Fig. 6, Additional file 2: Table S6; Itoh et al. 2017). RS content of un-mashed cooked rice can also be influenced by the grain size, degree of polishing, and amount of water added for cooking, however, this is likely because of earlier flowering and consequently higher temperature during seed development, which ensured an optimum temperature for RS biosynthesis.

Although RS is simply defined as starch that is not easily broken by digestive enzymes, increasing evidence shows that RS structure differs among different mutant rice lines (Miura et al. 2021). Structural analysis of residual starch after degradation by digestive enzymes suggests that fewer, but longer (DP > 15), amylopectin branches and higher amylose content correlate with greater resistance to degradation by digestive enzymes. This is because neighboring glucans form double helices, which, even though imperfect, make the starch less digestible (Miura et al. 2021). The content of longer glucans in residual starch is higher in mashed cooked rice than in raw rice flour because retrograded amylopectin likely forms double helices with nearby branches as well as with amylose, and becomes less degradable by digestive enzymes (Miura et al. 2021). When improving starch for health benefit, there are a couple of things that need to be considered. The initial starch structure will influence the rate of glucose release into the blood stream. Therefore, increasing the ratio of slowly digestible starch, in addition to RS, might be beneficial in preventing sudden postprandial glycemic responses. RS consisted with residual glucan after digestion can serve as a prebiotic, like fiber, and can enhance the production of short chain fatty acids by clonal flora, thus preventing colon cancer and allergies. The effects of different RS types with different structures on human health, and those of different processing methods on RS contents of food products remain to be elucidated.

## Conclusions

In the present study, RS-rich large-grain rice lines were successfully generated by backcrossing *be2b* and *ss3a be2b* lines, possessing active-type SSIIa and/or high GBSSI levels, with the elite rice cultivar, Akita 63. The seed weight of these lines increased to levels similar to or greater than that of WT *japonica* rice, and increased up to 190% compared with the seed weight before backcrossing. Presence of active-type SSIIa and/or high GBSSI levels increased the RS content of these lines further. Additionally, the RS contents of un-mashed cooked rice of these lines were high (27–35%) after backcrossing, whereas that of the WT was negligible (0.4%). In conclusion, to increase the RS content of mashed cooked rice and gelatinized rice flour, increasing GBSSI levels by introducing high-expressing *GBSS1* allele in the absence of BEIIb might be sufficient. However, increasing the RS content of raw rice flour in the absence of SSIIIa and BEIIb can be achieved by introducing both active-type *SS2a* and high-expressing *GBSS1* alleles, although the introduction of one of them alone can improve the RS content of mashed cooked rice and gelatinized rice flour. Furthermore, backcrossing of high RS lines improved their agricultural traits, without compromising the RS content. Overall, the present study provides important information for breeding new rice lines with improved agricultural traits and health benefits.

## Materials and Methods

### Plant Materials

Rice (*Oryza sativa* L.) *be2b* single mutant, EM10 (*ss2a*^*L*^* SS3a gbss1*^*L*^* be2b*), was previously isolated from *N*-methyl-*N*-nitrosourea (NMU) mutagenized populations of the *japonica* WT cultivar, Kinmaze (*ss2a*^*L*^* SS3a gbss1*^*L*^* BE2b*) (Yano et al. 1985; Nishi et al. 2001). EM10 harbors a mutation at the last nucleotide of *BEIIb* intron 9, which inhibits splicing, resulting in no protein production (Nagamatsu et al. 2022). The *ss3a be2b* mutant, #4019 (*ss2a*^*L*^* ss3a gbss1*^*L*^* be2b*), was generated by crossing *ss3a* mutant (e1; *ss2a*^*L*^* ss3a gbss1*^*L*^* BE2b*), a *Tos17* insertional mutant of Nipponbare, with *be2b* mutant, EM10 (Asai et al. 2014)*.* Line e1 harbors *Tos17* insertion in the first exon of *SSIIIa*, and does not produce the SSIIIa protein (Fujita et al. 2007). To introduce *SS2a* and/or *GBSS1* alleles, *be2b* or *ss3a be2b* were crossed with the *indica* rice cultivar, Kasalath (*SS2a SS3a GBSS1 BE2b*), and named #1203 (Itoh et al. 2017) or #1206, respectively. The #1206 line with active-type SSIIa and high levels of GBSSI, #1206C (*SS2a ss3a GBSS1 be2b*), was then crossed with the high-yielding elite *japonica* rice, Akita 63 (*ss2a*^*L*^* SS3a gbss1*^*L*^* BE2b*) (Makino et al. 2020). The resulting F_1_ seedlings were grown and self-pollinated to obtain the F_2_ progeny. DNA was isolated from the F_2_ seedlings, and genotyping was performed as described previously (Fujita et al. 2007; Crofts et al. 2017; Itoh et al. 2017). The following homozygous plants were isolated: #1203A (BC_3_), #1203B (BC_3_), and #1203C (BC_3_); two lines of #1203B (BC_3_), which were designated as #1203B (BC_3_)-1 and #1203B (BC_3_)-2; two lines of #1203C (BC_3_), which were designated as #1203C (BC_3_)-1 and #1203C (BC_3_)-2; #1206A (BC_3_), #1206B (BC_3_), and #1206C (BC_3_). The genotypes of lines used in this study are summarized in Table 1. F_3_ and F_4_ seeds were used for subsequent analyses. All rice lines were grown in an experimental paddy field of Akita Prefectural University during the summer months under natural light conditions.

### Western Blotting

Proteins were fractionated as described previously (Asai et al. 2014), with slight modifications. Briefly, mature rice grains were ground to a fine powder. SP + LBP was extracted using 10 volumes (w/v) of buffer containing 125 mM Tris–HCl (pH 6.8), 8 M urea, 4% (w/v) sodium dodecyl sulfate (SDS), and 5% (v/v) β-mercaptoethanol. After centrifugation, the supernatant was supplemented with half volume of 3× SDS-sample buffer containing 0.1 M Tris–HCl (pH 6.8), 10% SDS, 12% β-mercaptoethanol, 20% glycerol, and 0.2% bromophenol blue, and boiled for 5 min. The residual starch pellet obtained after extracting SP + LBP was washed with 1 mL of the buffer containing 125 mM Tris–HCl (pH 6.8), 4% (w/v) SDS, and 5% (v/v) β-mercaptoethanol. TBP were extracted overnight at room temperature using 15 volumes (w/v) of buffer containing 125 mM Tris–HCl (pH 6.8), 8 M urea, 4% (w/v) SDS, 5% (v/v) β-mercaptoethanol, and 0.05% (w/v) bromophenol blue. After centrifugation, supernatants were used. All protein samples were separated by SDS–polyacrylamide gel electrophoresis on 7.5% acrylamide gel, and blotted onto a membrane. Membranes were incubated with following primary antibodies: anti-SSIIa (1:1000 dilution; Crofts et al. 2015), anti-SSIIIa (1:1000; Crofts et al. 2012), anti-GBSSI (1:5000; Fujita et al. 2006), and anti-BEIIb (1:5000; Nishi et al. 2001). Subsequently, secondary antibody incubation and protein detection were performed as described previously (Crofts et al. 2015).

### Seed Morphology

Seed morphology of brown rice was observed using a stereomicroscope (SZX7-ILST-0 (SP), OLYMPUS, Tokyo, Japan), with light sources above and underneath. Photos were taken using a digital camera, as described previously (Miura et al. 2018).

### Amylose Content

Starch was purified from polished rice grains using the cold-alkaline method, as described previously (Yamamoto et al. 1973, 1981). Starch was debranched using *Pseudomonas* isoamylase (Hayashibara, Okayama, Japan) and analyzed by gel filtration chromatography (Toyopearl HW-55S and HW-50S×3; Tosoh, Tokyo, Japan) (Horibata et al. 2004; Fujita et al. 2007; Toyosawa et al. 2016). Amylose (fraction I), long amylopectin chains (fraction II), short amylopectin chains (fraction III), and apparent amylose content were quantified as described previously (Horibata et al. 2004; Fujita et al. 2007; Toyosawa et al. 2016).

### Amylopectin Structure Analysis

Starch was isolated from mature rice seeds, and debranched using *Pseudomonas* isoamylase (Hayashibara, Okayama, Japan). Then, the debranched starch was fluorescently labeled and analyzed by capillary electrophoresis (P/ACE MDQ Plus Carbohydrate System, AB Sciex, Framingham, Ma, USA), as described by Fujita et al. (2001).

### RS Content Measurement

RS contents of polished rice in different forms (mashed cooked rice, un-mashed cooked rice, raw rice flour, and gelatinized rice flour) were determined as described previously (Miura et al. 2021).

### Gelatinization Temperature

The thermal properties of purified starch were analyzed by differential scanning calorimetry (Seiko Instrument 6100, Chiba, Japan), as described previously (Fujita et al. 2003, 2006).

### Data Analyses

Seed weight, amylose content, thermal properties, and RS contents were statistically analyzed by Tukey–Kramer method (*P* < 0.05).

## Supplementary Information

**Additional file 1: Fig. S1**. Pedigree of #1203, #1206, #1203 (BC_3_), and #1206 (BC_3_) lines. **Fig. S2.** Elution profiles of debranched endosperm starch separated by gel filtration chromatography. **Fig. S3.** Comparison of amylopectin structure before and after backcrossing. **Fig. S4.** Differences in amylopectin structure between #1203 (BC_3_) lines and Akita 63 to determine the effect of BEIIb loss in addition to the effects of SSIIa and/or GBSSI. **Fig. S5.** Differences in amylopectin structure showing the effect of active SSIIa. **Fig. S6.** Differences in amylopectin structure between #1206 (BC_3_) lines and Akita 63 to determine the effect of the loss of SSIIIa and BEIIb in addition to the effects of SSIIa and/or GBSSI. **Fig. S7.** Differences in amylopectin structure showing the effect of high expression level of GBSSI. **Fig. S8.** Typical differential scanning calorimetry profiles of rice lines showing peak gelatinization temperature.

**Additional file 2: Table S1**. Genotypes and grain weight of rice lines before backcrossing. **Table S2.** Flowering dates of rice lines and cumulative temperature before and after backcrossing. **Table S3.** Apparent amylose, long amylopectin chain, and short amylopectin contents and short-to-long amylopectin chain ratio in the endosperm starch of rice lines after backcrossing measured by gel filtration chromatography using debranched starch. **Table S4.** Apparent amylose, long amylopectin chain, and short amylopectin contents and short-to-long amylopectin chain ratio in the endosperm starch of rice lines before backcrossing measured by gel filtration chromatography using debranched starch. **Table S5.** Differential scanning calorimetry analysis of the thermal properties of starch in rice lines before backcrossing. **Table S6.** RS contents of raw and cooked rice flour and un-mashed and mashed cooked rice grains.

## Data Availability

All data generated or analyzed in this study are included in this published. article and its additional information files.

## Abbreviations

BE: Starch branching enzyme
DBE: Debranching enzyme
DP: Degree of polymerization
DSC: Differential scanning calorimetry
Fr: Fraction
GBSSI: Granule-bound starch synthase I
LBP: Proteins loosely-bound to starch granule
RS: Resistant starch
SDS: Sodium dodecyl sulfate
SP: Soluble protein
SS: Starch synthase
TBP: Proteins tightly-bound to starch granule
WT: Wild-type
