# Supplementary material for: Improving Agricultural Traits While Maintaining High Resistant Starch Content in Rice
Source: Rice (N Y). 2022 Jun 4;15:28. doi: 10.1186/s12284-022-00573-5 (PMC9167398; doi:10.1186/s12284-022-00573-5)
Supplement: Supplementary file 1 — Additional file 1: Fig. S1. Pedigree of #1203, #1206, #1203 (BC3), and #1206 (BC3) lines. Fig. S2. Elution profiles of debranched endosperm starch separated by gel filtration chromatography. Fig. S3. Comparison of amylopectin structure before and after backcrossing. Fig. S4. Differences in amylopectin structure between #1203 (BC3) lines and Akita 63 to determine the effect of BEIIb loss in addition to the effects of SSIIa and/or GBSSI. Fig. S5. Differences in amylopectin structure showing the effect of active SSIIa. Fig. S6. Differences in amylopectin structure between #1206 (BC3) lines and Akita 63 to determine the effect of the loss of SSIIIa and BEIIb in addition to the effects of SSIIa and/or GBSSI. Fig. S7. Differences in amylopectin structure showing the effect of high expression level of GBSSI. Fig. S8. Typical differential scanning calorimetry profiles of rice lines showing peak gelatinization temperature. [file 12284_2022_573_MOESM1_ESM.pdf]

(A) Before backcrossing

P

EM10: $ss2a^{L}$ *SS3a* $gbss1^{L}$ *be2b*

or

#4019: $ss2a^{L}$ *ss3a* $gbss1^{L}$ *be2b*

× Kasalath: *SS2a SS3a GBSS1 BE2b*

↓

F_1

SS2a $ss2a^{L}$ *SS3a SS3a GBSS1* $gbss1^{L}$ *BE2b be2b* × *SS2a* $ss2a^{L}$ *SS3a SS3a GBSS1* $gbss1^{L}$ *BE2b be2b*

or

SS2a $ss2a^{L}$ *SS3a ss3a GBSS1* $gbss1^{L}$ *BE2b be2b* × *SS2a* $ss2a^{L}$ *SS3a ss3a GBSS1* $gbss1^{L}$ *BE2b be2b*

↓

F_2 Screening of genotype by immunoblotting, PCR and dCAPs markers methods.

↓

F_3

#1203A: *SS2a SS3a* $gbss1^{L}$ *be2b*
#1203B: $ss2a^{L}$ *SS3a GBSS1 be2b**
#1203C: *SS2a SS3a GBSS1 be2b**

or

#1206A: *SS2a ss3a* $gbss1^{L}$ *be2b*
#1206B: $ss2a^{L}$ *ss3a GBSS1 be2b*
#1206C: *SS2a ss3a GBSS1 be2b*

Fig. S1A. Pedigree of #1203 and #1206 lines before backcrossing. *The lines previously described (Itoh et al. 2017).

(B) After backcrossing

P Akita 63: *ss2a*^{L} *SS3a gbss1*^{L} *BE2b* × #1206C: *SS2a ss3a GBSS1 be2b*

↓

F_1 Akita 63: *ss2a*^{L} *SS3a gbss1*^{L} *BE2b* × *SS2a ss2a*^{L} *SS3a ss3a GBSS1 gbss1*^{L} *BE2b be2b*

↓

F_2 Screening of genotype by immunoblotting, PCR and dCAPs markers methods.

(Back cross ×3)

↓

F_3

#1203A (BC_3): *SS2a SS3a gbss1*^{L} *be2b*
#1203B (BC_3): *ss2a*^{L} *SS3a GBSS1 be2b*
#1203C (BC_3): *SS2a SS3a GBSS1 be2b*

or

#1206A (BC_3): *SS2a ss3a gbss1*^{L} *be2b*
#1206B (BC_3): *ss2a*^{L} *ss3a GBSS1 be2b*
#1206C (BC_3): *SS2a ss3a GBSS1 be2b*

Fig. S1B. Pedigree of #1203 (BC_3) and #1206 (BC_3) lines after backcrossing.

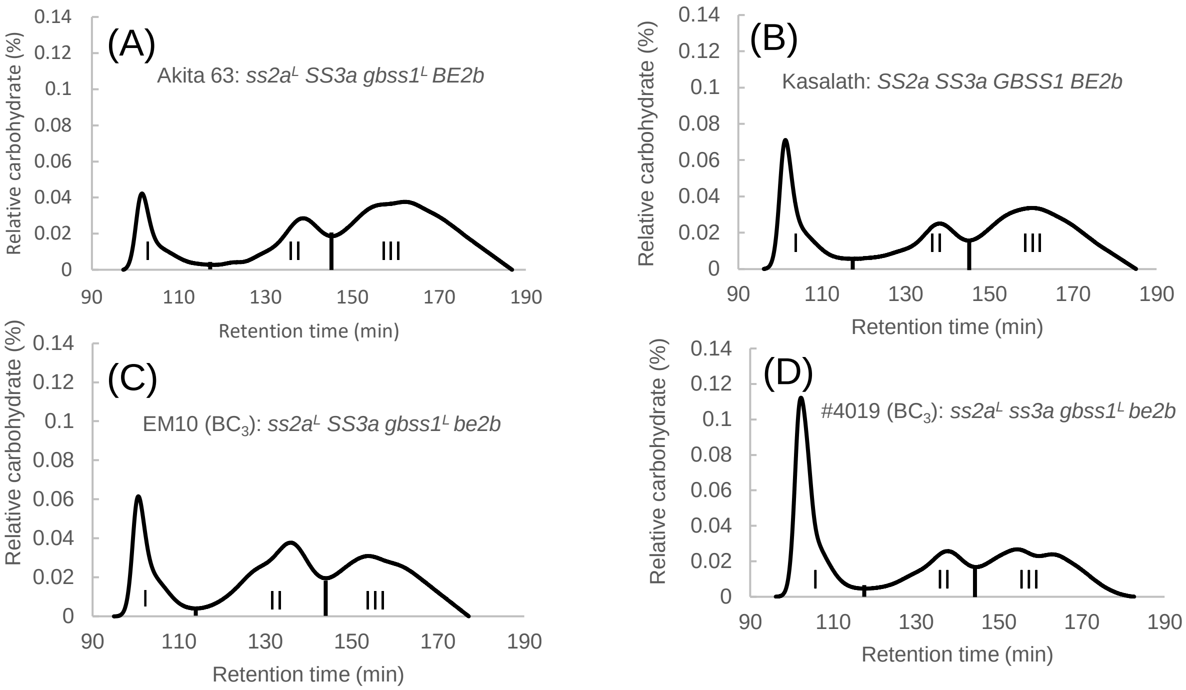

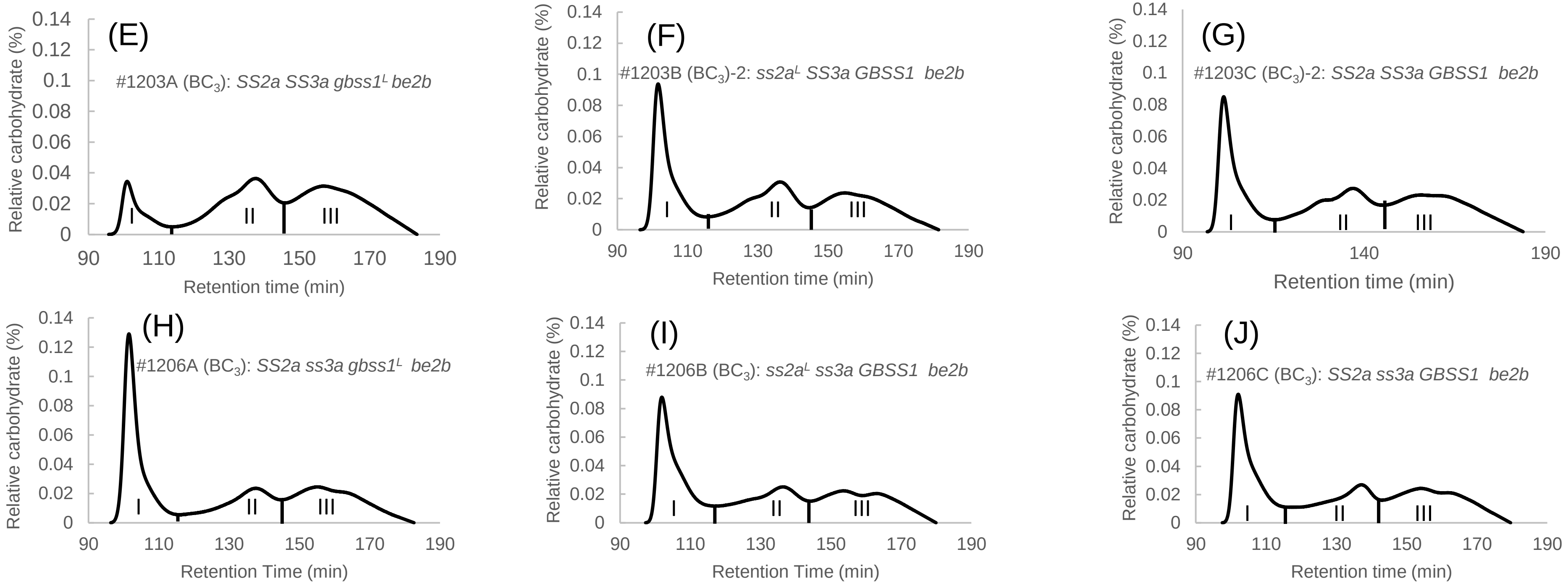

Fig. S2. Elution profiles of debranched endosperm starch separated by gel filtration chromatography.
Fraction I contains amylose and extra-long chains of amylopectin; fraction II contains long chains of amylopectin; and fraction III contains short chains of amylopectin.

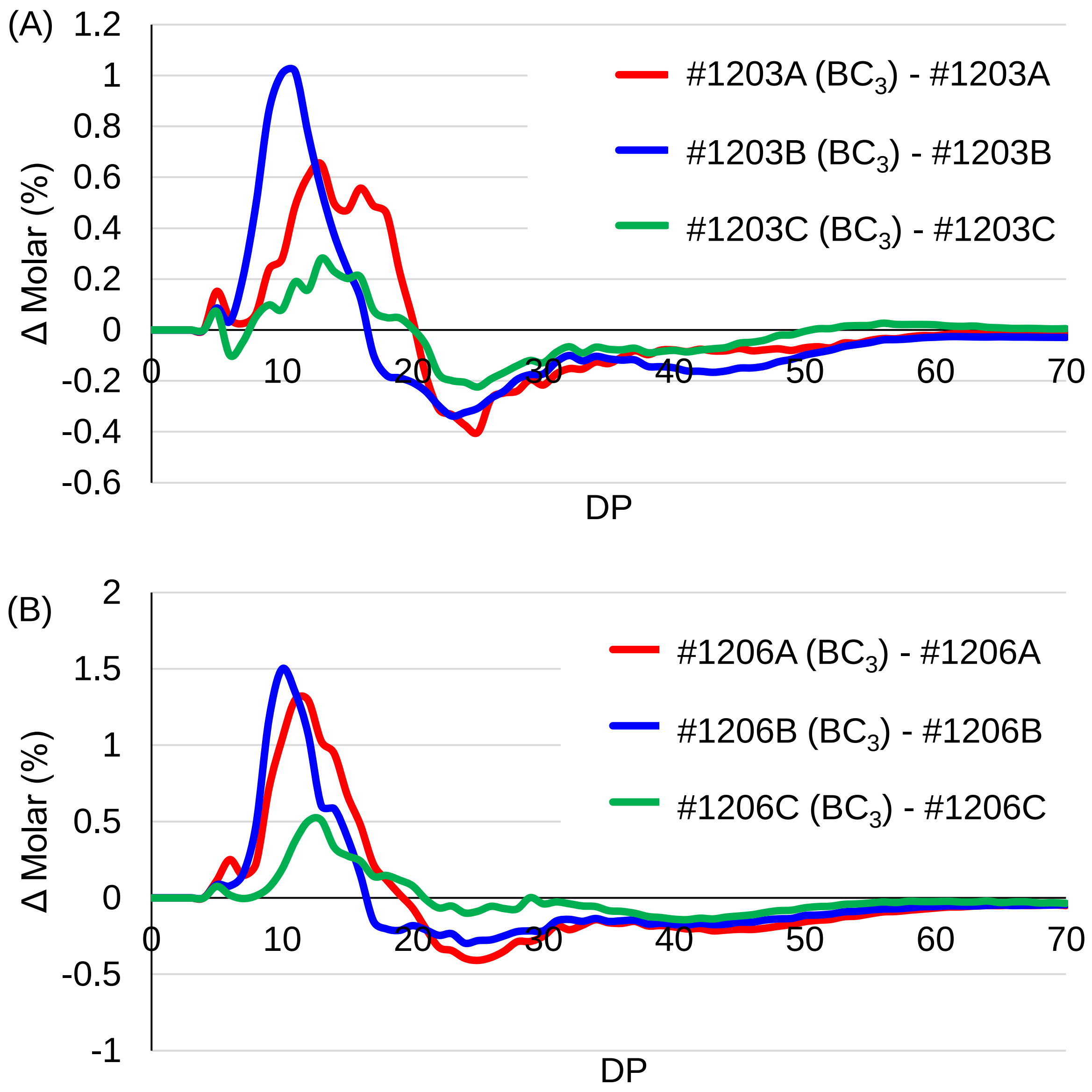

Fig. S3. Comparison of amylopectin structure before and after backcrossing. Differences are shown as Δ Molar %, and the value was calculated by subtracting the pattern of before from after backcrossing, as indicated.

(A) #1203A: *SS2a SS3a gbss1^L be2b*

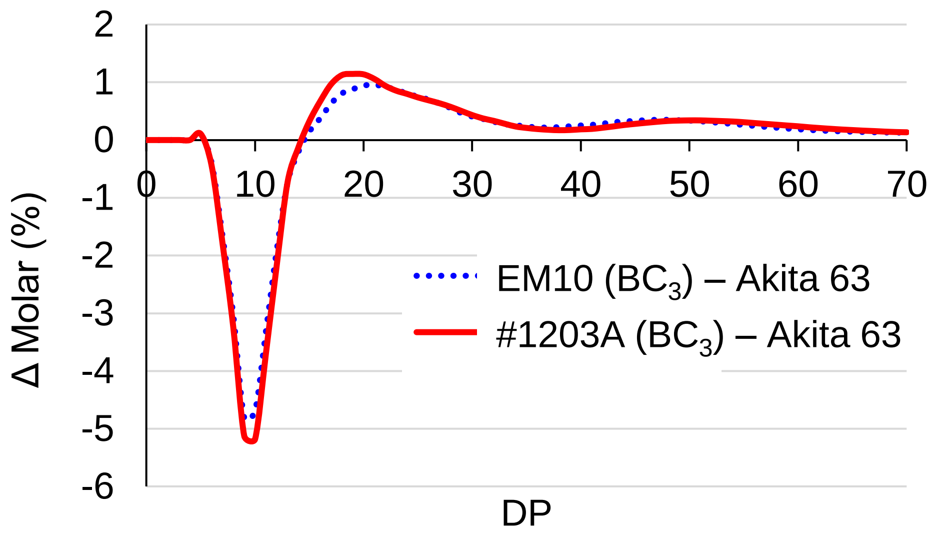

(B) #1203B: *ss2a^L SS3a GBSS1 be2b*

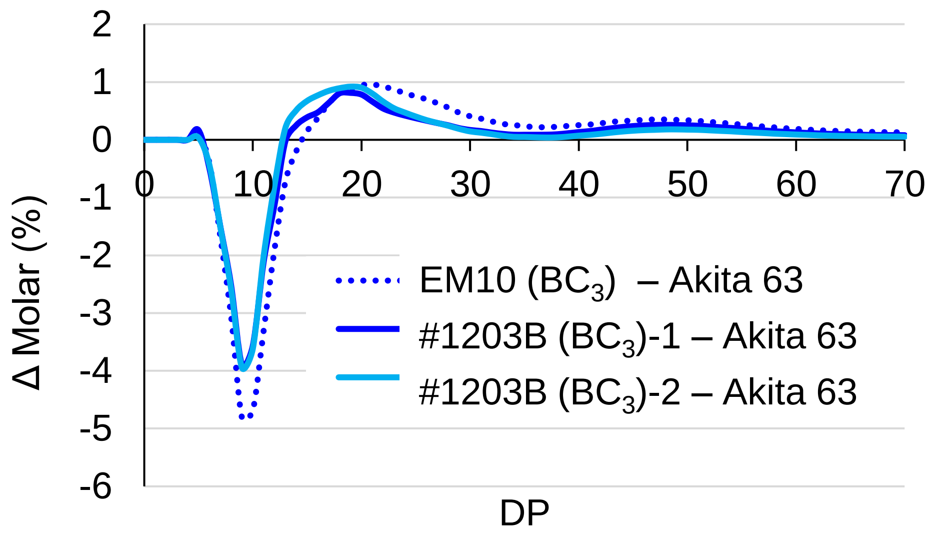

(C) #1203C: *SS2a SS3a GBSS1 be2b*

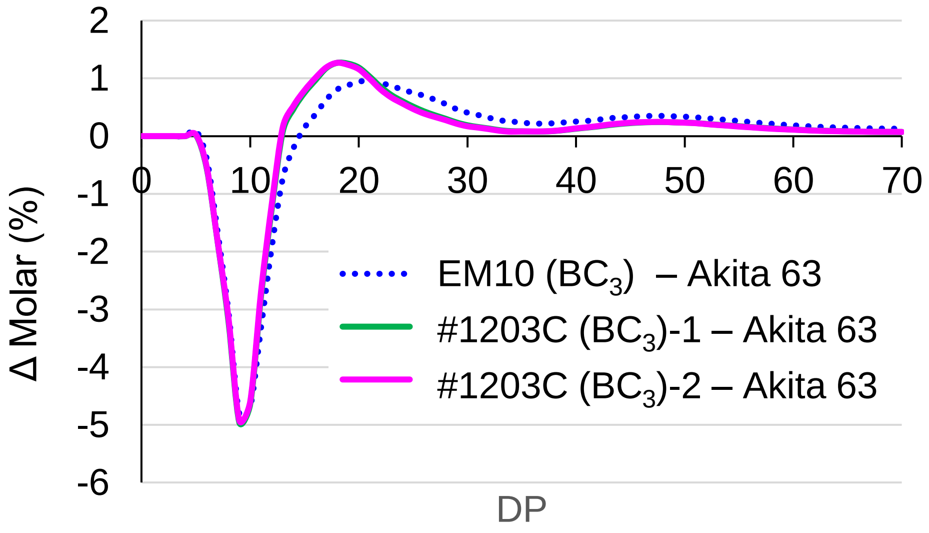

Fig. S4. Differences in amylopectin structure between #1203 (BC_3) lines and Akita 63 to determine the effect of BEIIb loss in addition to the effects of SSIIa and/or GBSSI.
Differences are shown as Δ Molar %, and the value was calculated by subtracting the pattern of Akita 63 from each line, as indicated.

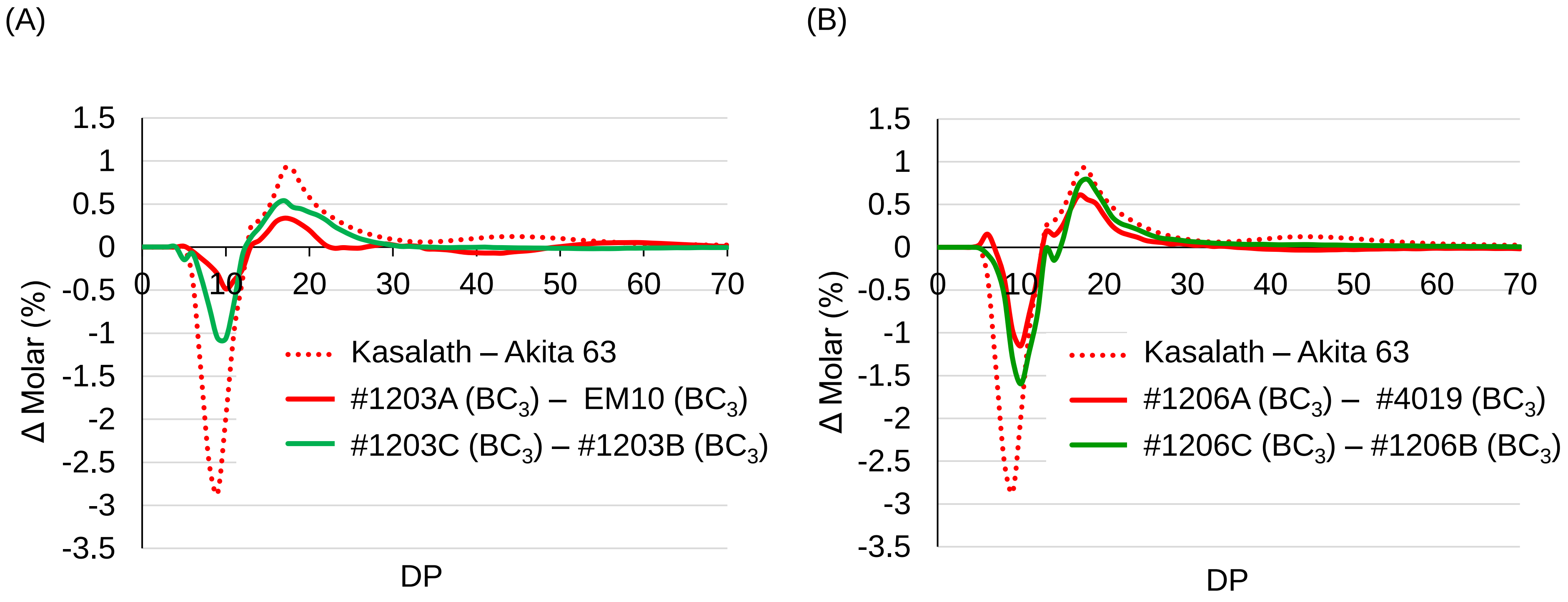

Fig. S5. Differences in amylopectin structure showing the effect of active SSIIa.
Differences are shown as Δ Molar %, and the value was calculated by subtracting the pattern of less-active SSIIa (*ss2a*^{L}) from active-SSIIa line (*SS2a*), as indicated.

(A) #1206A (BC_3): *SS2a ss3a gbss1^L be2b*

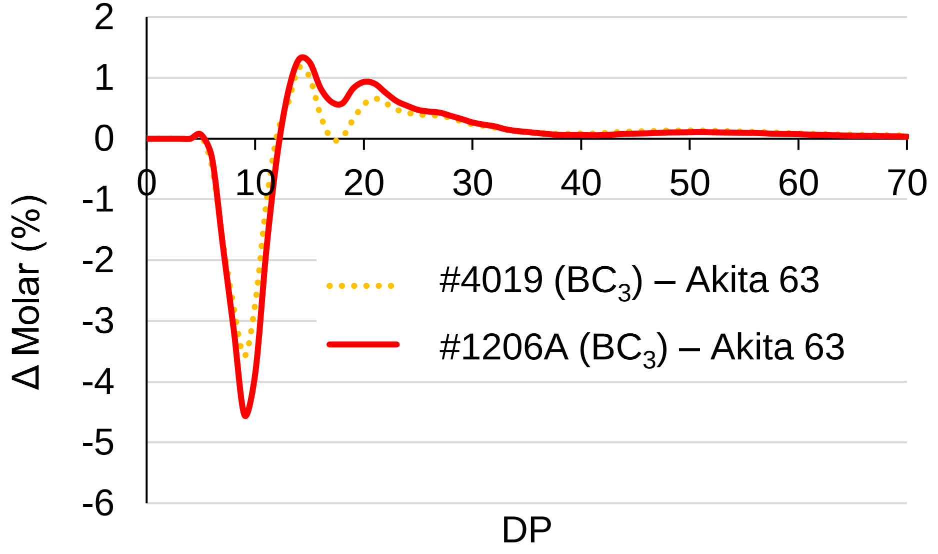

(B) #1206B (BC_3): *ss2a^L ss3a GBSS1 be2b*

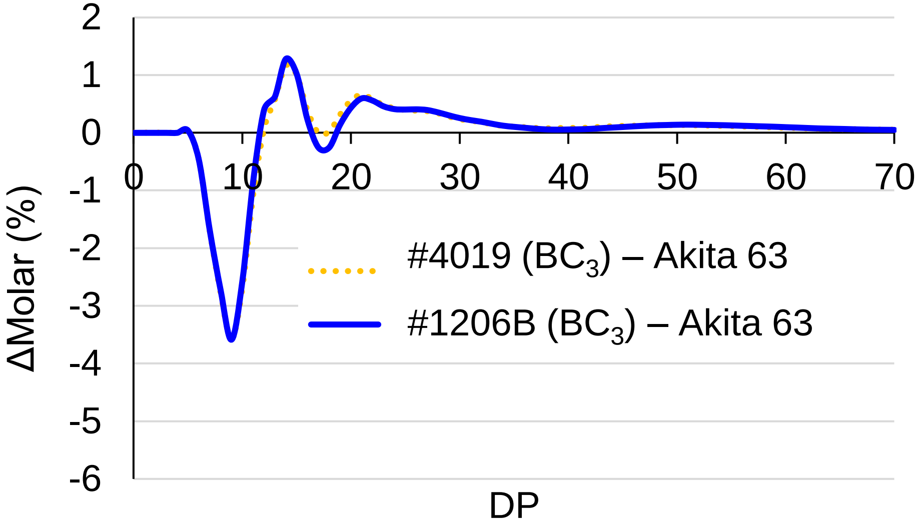

(C) #1206C (BC_3): *SS2a ss3a GBSS1 be2b*

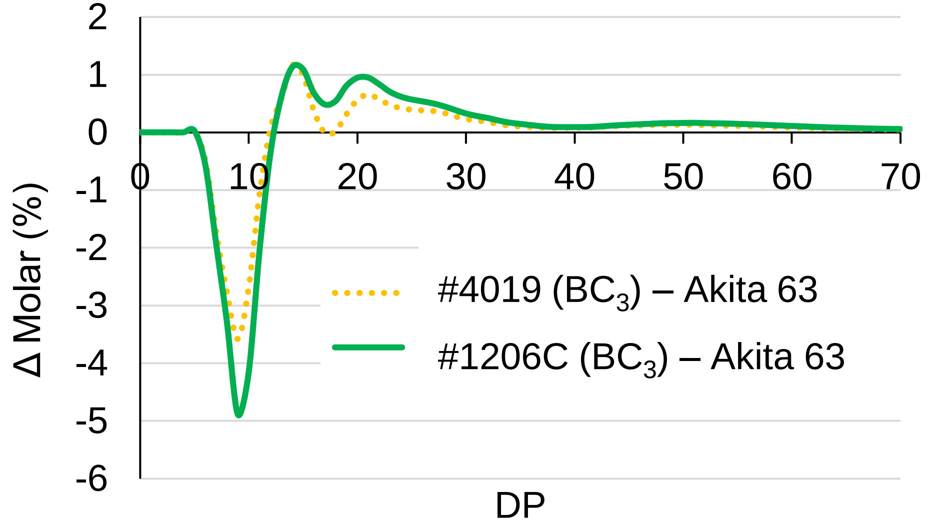

Fig. S6. Differences in amylopectin structure between #1206 (BC_3) lines and Akita 63 to determine the effect of the loss of SSIIIa and BEIIb in addition to the effects of SSIIa and/or GBSSI.

Differences are shown as Δ Molar %, and the value was calculated by subtracting the pattern of WT from each mutant line, as indicated.

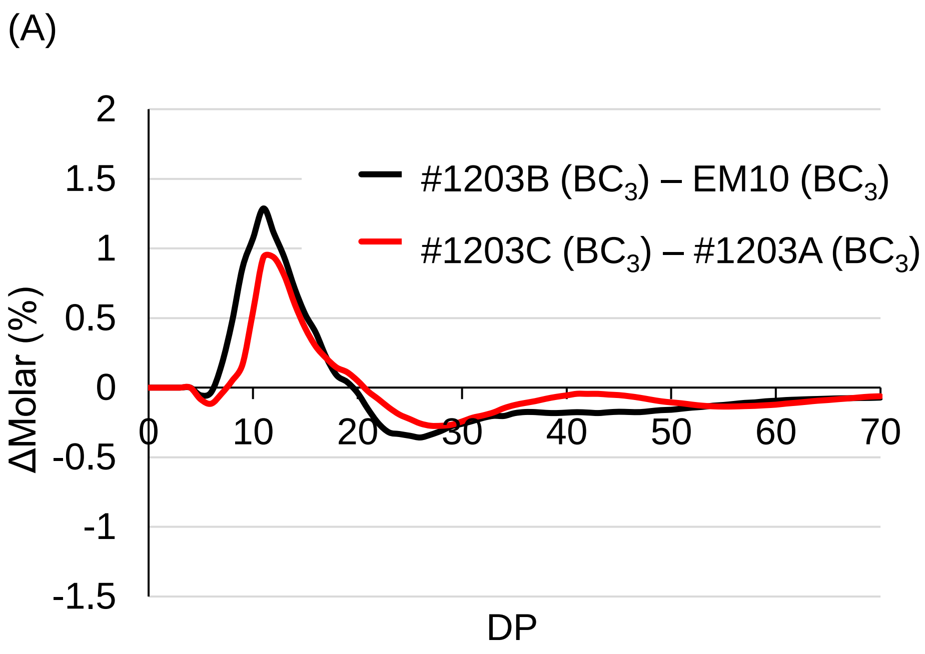

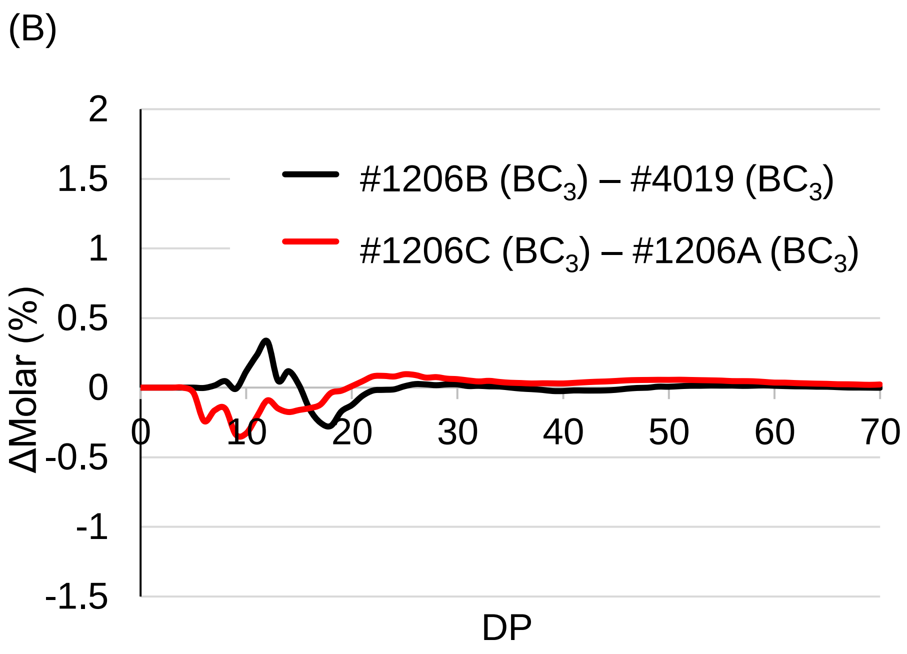

Fig. S7. Differences in amylopectin structure showing the effect of high expression level of GBSSI. Differences are shown as Δ Molar %, and the value was calculated by subtracting the pattern of low-expression GBSSI (*gbss1*^{L}) from high-expression GBSSI (*GBSS1*) line, as indicated. Black line indicates a theoretical value calculated by adding the effects of the loss of BEI alone and BEIIb alone.

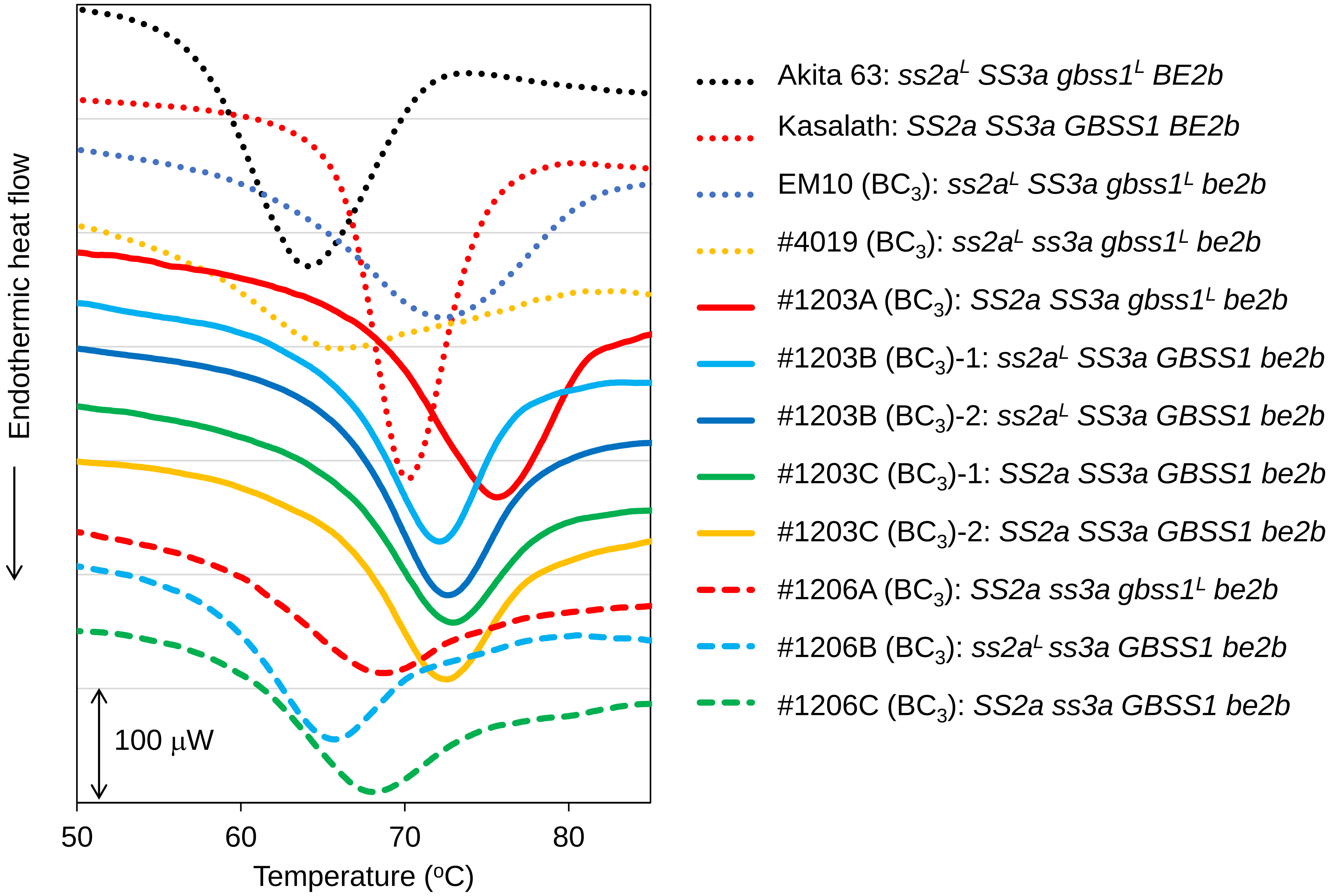

Fig. S8. Typical differential scanning calorimetry profiles of rice lines showing peak gelatinization temperature. Data represents one of at least three independent replications using purified starch.
