# Supplementary material for: Improving Agricultural Traits While Maintaining High Resistant Starch Content in Rice
Source: Rice (N Y). 2022 Jun 4;15:28. doi: 10.1186/s12284-022-00573-5 (PMC9167398; doi:10.1186/s12284-022-00573-5)
Supplement: Supplementary file 2 — Additional file 2: Table S1. Genotypes and grain weight of rice lines before backcrossing. Table S2. Flowering dates of rice lines and cumulative temperature before and after backcrossing. Table S3. Apparent amylose, long amylopectin chain, and short amylopectin contents and short-to-long amylopectin chain ratio in the endosperm starch of rice lines after backcrossing measured by gel filtration chromatography using debranched starch. Table S4. Apparent amylose, long amylopectin chain, and short amylopectin contents and short-to-long amylopectin chain ratio in the endosperm starch of rice lines before backcrossing measured by gel filtration chromatography using debranched starch. Table S5. Differential scanning calorimetry analysis of the thermal properties of starch in rice lines before backcrossing. Table S6. RS contents of raw and cooked rice flour and un-mashed and mashed cooked rice grains. [file 12284_2022_573_MOESM2_ESM.pdf]

Table S1. Genotypes and grain weight of rice lines before backcrossing.

Line	Genotype	Grain weight (mg)[1]	Grain weight (%)
Kasalath	*SS2a SS3a GBSS1 BE2b*	15.4 ± 0.2 cdef	81
Kinmaze	*ss2a^L SS3a gbss1^L BE2b*	19.0 ± 0.3 ab	100
Nipponbare	*ss2a^L SS3a gbss1^L BE2b*	20.0 ± 0.3 a	105
e1	*ss2a^L ss3a gbss1^L BE2b*	18.7 ± 0.6 ab	98
EM10	*ss2a^L SS3a gbss1^L be2b*	10.7 ± 0.3 ij	56
#4019	*ss2a^L ss3a gbss1^L be2b*	15.9 ± 0.2 cde	83
#1203A12	*SS2a SS3a gbss1^L be2b*	9.7 ± 0.7 j	51
#1203A14	*SS2a SS3a gbss1^L be2b*	9.1 ± 0.3 j	48
#1203B21*	*ss2a^L SS3a GBSS1 be2b*	15.1 ± 0.4 ef	80
#1203B23*	*ss2a^L SS3a GBSS1 be2b*	15.5 ± 0.2 cdef	81
#1203C11*	*SS2a SS3a GBSS1 be2b*	15.1 ± 0.2 ef	79
#1203C32*	*SS2a SS3a GBSS1 be2b*	15.7 ± 0.3 cde	82
#1206A11	*SS2a ss3a gbss1^L be2b*	13.5 ± 0.2 fgh	71
#1206A51	*SS2a ss3a gbss1^L be2b*	12.9 ± 0.3 gh	68
#1206B92	*ss2a^L ss3a GBSS1 be2b*	17.4 ± 0.6 bc	92
#1206B93	*ss2a^L ss3a GBSS1 be2b*	17.4 ± 0.3 bcd	91
#1206C71	*SS2a ss3a GBSS1 be2b*	15.3 ± 0.4 def	80
#1206C81	*SS2a ss3a GBSS1 be2b*	12.6 ± 0.3 hi	66
#1206C83	*SS2a ss3a GBSS1 be2b*	14.9 ± 0.4 efg	78

[1]The weight (mg) of one dehulled mature grain of each rice line is presented as mean ± standard error (SE; *n* = 20) as well as a percentage of the seed weight of the wild-type (WT) cultivar, Kinmaze. Different lowercase letters indicate significant differences among rice genotypes (*P* <0.05; Tukey-Kramer method). *Data from Itoh et al. 2017.

Table S2. Flowering dates of rice lines and cumulative temperature before and after backcrossing.

Line	Genotype	2020 Flowering date	2020 Cumulative temperature
Akita 63	*ss2a*^{L} *SS3a gbss1*^{L} *BE2b*	8/2	680.8
Kasalath	*SS2a SS3a GBSS1 BE2b*	8/12	698.0
Kinmaze	*ss2a*^{L} *SS3a gbss1*^{L} *BE2b*	-	-
Nipponbare	*ss2a*^{L} *SS3a gbss1*^{L} *BE2b*	-	-
e1	*ss2a*^{L} *ss3a gbss1*^{L} *BE2b*	-	-
After backcrossing			
EM10 (BC$_3$)	*ss2a*^{L} *SS3a gbss1*^{L} *be2b*	7/30	685.5
#4019 (BC$_3$)	*ss2a*^{L} *ss3a gbss1*^{L} *be2b*	7/31	685.4
#1203A (BC$_3$)	*SS2a SS3a gbss1*^{L} *be2b*	8/5	693.3
#1203B (BC$_3$)	*ss2a*^{L} *SS3a GBSS1 be2b*	8/5	693.3
#1203C (BC$_3$)	*SS2a SS3a GBSS1 be2b*	8/3	684.2
#1206A (BC$_3$)	*SS2a ss3a gbss1*^{L} *be2b*	8/2	680.8
#1206B (BC$_3$)	*ss2a*^{L} *ss3a GBSS1 be2b*	8/2	680.8
#1206C (BC$_3$)	*SS2a ss3a GBSS1 be2b*	8/2	680.8

Line	2015 Flowering date	2015 Cumulative temperature	2016 Flowering date	2016 Cumulative temperature
Akita 63	-	-	-	-
Kasalath	8/5	615.7	8/6	682.0
Kinmaze	9/5	488.7	8/25	595.4
Nipponbare	8/26	536.2	8/25	595.4
e1	8/27	533.8	8/25	595.4
Before backcrossing				
EM10	9/5	488.7	9/1	549.6
#4019	8/26	536.2	8/25	595.4
#1203A12	8/15	568.8	8/17	640.0
#1203A14	8/15	568.8	8/17	640.0
#1203B21	9/25	363.6	9/15	452.2
#1203B23	8/30	519.2	8/25	595.4
#1203C11	8/15	568.8	8/17	640.0
#1203C32	8/8	596.0	8/17	640.0
#1206A11	8/8	596.0	8/17	640.0
#1206A51	8/21	548.2	8/17	640.0
#1206B92	8/29	524.5	8/27	588.0
#1206B93	8/25	537.4	8/27	588.0
#1206C71	8/8	596.0	8/18	634.7
#1206C81	8/15	568.8	8/18	634.7
#1206C83	8/24	539.1	8/18	634.7

Cumulative temperature between 5 and 30 days after flowering were calculated according to the data released by Japan Metrological Agency.

Table S3. Apparent amylose content, content of long and short amylopectin chains, and the ratio of short to long amylopectin chains in endosperm after backcrossing measured by gel filtration chromatography using debranched starch.

Line	Genotype	Fr. I (%)	Fr. II (%)	Fr. III (%)	III/II
Akita 63	*ss2a*^{L} *SS3a gbss1*^{L}*BE2b*	16.2 ± 0.4e	23.1 ± 0.1ef	60.7 ± 0.5a	2.6 ± 0.0a
Kasalath	*SS2a SS3a GBSS1 BE2b*	26.7 ± 0.5d	22.3 ± 0.5f	51.0 ± 0.5b	2.3 ± 0.1a
EM10 (BC_3)	*ss2a*^{L} *SS3a gbss1*^{L} *be2b*	20.7 ± 0.5d	37.4 ± 0.7ab	41.9 ± 1.1c	1.1 ± 0.1de
#4019 (BC_3)	*ss2a*^{L} *ss3a gbss1*^{L} *be2b*	39.0 ± 0.5ab	22.9 ± 0.3ef	38.1 ± 0.2c	1.7 ± 0.0b
#1203A (BC_3)	*SS2a SS3a gbss1*^{L} *be2b*	14.6 ± 0.2e	41.8 ± 0.1a	43.6 ± 0.2c	1.0 ± 0.0de
#1203B (BC_3)-1	*ss2a*^{L} *SS3a GBSS1 be2b*	34.9 ± 0.4bc	34.4 ± 0.2bc	30.7 ± 0.5e	0.9 ± 0.0e
#1203B (BC_3)-2	*ss2a*^{L} *SS3a GBSS1 be2b*	34.5 ± 0.4c	32.7 ± 0.1c	32.8 ± 0.3de	1.0 ± 0.0de
#1203C (BC_3)-1	*SS2a SS3a GBSS1 be2b*	35.2 ± 0.2bc	32.8 ± 0.2c	32.0 ± 0.0de	1.0 ± 0.0e
#1203C (BC_3)-2	*SS2a SS3a GBSS1 be2b*	35.1 ± 0.5bc	33.2 ± 0.3c	31.7 ± 0.6de	1.0 ± 0.1e
#1206A (BC_3)	*SS2a ss3a gbss1*^{L} *be2b*	41.9 ± 0.4a	22.9 ± 0.6ef	35.2 ± 0.3de	1.5 ± 0.0bc
#1206B (BC_3)	*ss2a*^{L} *ss3a GBSS1 be2b*	38.1 ± 0.7abc	28.0 ± 0.8d	33.9 ± 0.1de	1.2 ± 0.0cde
#1206C (BC_3)	*SS2a ss3a GBSS1 be2b*	36.6 ± 0.4bc	27.0 ± 0.1de	36.4 ± 0.5cd	1.3 ± 0.0bcd

Data represent mean ± standard error (SE; n = 3). Fraction I contains amylose and extra-long chains of amylopectin; fraction II contains long chains of amylopectin; and fraction III contains short chains of amylopectin. Significant differences are indicated with different lowercase letters ($P < 0.05$; Tukey-Kramer method).

Table S4. Apparent amylose content, content of long and short amylopectin chains, and the ratio of short to long amylopectin chains in endosperm starch before backcrossing measured by gel filtration chromatography using debranched starch.

Line	Genotype	Fr. I (%)	Fr. II (%)	Fr. III (%)	III/II
Kasalath	*SS2a SS3a GBSS1 BE2b*	26.8 ± 0.7de	21.9 ± 0.5fgh	51.3 ± 1.2ab	2.3 ± 0.1c
Kinmaze	*ss2a^L SS3a gbss1^L BE2b*	20.7 ± 0.2ef	18.9 ± 0.2h	60.4 ± 0.2a	3.2 ± 0.1b
Nipponbare	*ss2a^L SS3a gbss1^L BE2b*	19.2 ± 0.5ef	21.1 ± 0.5gh	59.7 ± 1.0a	2.8 ± 0.1bc
e1	*ss2a^L ss3a gbss1^L BE2b*	31.7 ± 0.4cd	13.5 ± 0.3i	54.8 ± 0.2ab	4.1 ± 0.1a
EM10	*ss2a^L SS3a gbss1^L be2b*	27.3 ± 0.8de	37.5 ± 0.6abc	35.2 ± 0.3c	0.9 ± 0.0efg
#4019	*ss2a^L ss3a gbss1^L be2b*	44.6 ± 0.2ab	22.3 ± 0.3fgh	33.1 ± 0.4c	1.5 ± 0.0d
#1203A12	*SS2a SS3a gbss1^L be2b*	16.4 ± 0.5f	37.6 ± 0.4ab	46.0 ± 0.8b	1.2 ± 0.0defg
#1203A14	*SS2a SS3a gbss1^L be2b*	13.4 ± 1.9f	40.1 ± 0.5a	46.5 ± 2.7b	1.2 ± 0.1defg
#1203B21	*ss2a^L SS3a GBSS1 be2b*	36.6 ± 0.0bc	32.8 ± 0.2cd	30.6 ± 0.2c	0.9 ± 0.0efg
#1203B23	*ss2a^L SS3a GBSS1 be2b*	38.6 ± 0.2bc	33.2 ± 0.3bc	28.2 ± 0.1c	0.8 ± 0.0g
#1203C11	*SS2a SS3a GBSS1 be2b*	37.1 ± 0.3bc	33.6 ± 0.2bc	29.3 ± 0.4c	0.9 ± 0.0fg
#1203C32	*SS2a SS3a GBSS1 be2b*	36.6 ± 0.4bc	33.0 ± 0.3bc	30.4 ± 0.6c	0.9 ± 0.0efg
#1206A11	*SS2a ss3a gbss1^L be2b*	41.1 ± 0.9ab	24.0 ± 0.3fg	34.9 ± 0.8c	1.4 ± 0.0de
#1206A51	*SS2a ss3a gbss1^L be2b*	47.9 ± 1.1a	21.7 ± 0.6fgh	30.4 ± 0.5c	1.4 ± 0.0def
#1206B92	*ss2a^L ss3a GBSS1 be2b*	40.3 ± 1.2abc	26.3 ± 0.2ef	33.4 ± 1.0c	1.3 ± 0.0defg
#1206B93	*ss2a^L ss3a GBSS1 be2b*	42.2 ± 0.6ab	25.3 ± 0.2efg	32.5 ± 0.3c	1.3 ± 0.0defg
#1206C71	*SS2a ss3a GBSS1 be2b*	44.5 ± 1.3ab	25.1 ± 0.6efg	30.4 ± 0.7c	1.2 ± 0.0defg
#1206C81	*SS2a ss3a GBSS1 be2b*	40.2 ± 0.6abc	28.1 ± 0.3de	31.7 ± 0.4c	1.1 ± 0.0defg
#1206C83	*SS2a ss3a GBSS1 be2b*	40.5 ±0.2 ab	27.6 ± 0.4e	31.9 ± 0.6c	1.2 ± 0.0defg

Data represent mean ± standard error (SE; n = 3). Fraction I contains amylose and extra-long chains of amylopectin, fraction II contains long chains of amylopectin, and fraction III contains short chains of amylopectin. Significant differences are indicated with different lowercase letters (P <0.05; Tukey-Kramer method).

Table S5. Differential scanning calorimetry analysis of the thermal properties of starch in rice lines before backcrossing.

Line		Genotype	T_O (°C)	T_P (°C)	T_C (°C)	ΔH (J/g)
’15	Kasalath	*SS2a SS3a GBSS1 BE2b*	63.3 ± 0.0b	67.8 ± 0.0e	72.1 ± 0.0g	15.8 ± 0.2ab
’15	Kinmaze	*ss2a^L SS3a gbss1^L BE2b*	45.8 ± 0.0m	53.6 ± 0.1m	60.0 ± 0.1m	12.4 ± 0.2hijk
’15	Nipponbare	*ss2a^L SS3a gbss1^L BE2b*	50.0 ± 0.1m	57.3 ± 0.0l	63.6 ± 0.2l	13.3 ± 0.3ijkl
’15	*e1*	*ss2a^L ss3a gbss1^L BE2b*	49.3 ± 0.3m	57.2 ± 0.0l	63.9 ± 0.2l	11.6 ± 0.5defgh
’15	*EM10*	*ss2a^L SS3a gbss1^L be2b*	56.8 ± 0.3ef	68.4 ± 0.1c	81.1 ± 0.1a	16.9 ± 0.3a
’15	*#4019*	*ss2a^L ss3a gbss1^L be2b*	53.3 ± 0.6kl	62.0 ± 0.1j	69.8 ± 0.7j	11.3 ± 1.3ijkl
’16	Kasalath	*SS2a SS3a GBSS1 BE2b*	65.7 ± 0.1a	69.2 ± 0.0b	73.3 ± 0.1e	16.2 ± 0.3ab
’16	Kinmaze	*ss2a^L SS3a gbss1^L BE2b*	53.7 ± 0.1jk	59.8 ± 0.0k	65.4 ± 0.1k	12.8 ± 0.4fghij
’16	*EM10*	*ss2a^L SS3a gbss1^L be2b*	57.4 ± 0.2e	68.1 ± 0.2cde	78.0 ± 0.4b	14.4 ± 0.2bcdef
’16	#1203A12	*SS2a SS3a gbss1^L be2b*	65.1 ± 0.5a	73.6 ± 0.1a	80.8 ± 0.2a	14.6 ± 0.6bcde
’16	#1203A14	*SS2a SS3a gbss1^L be2b*	65.5 ± 0.3a	73.8 ± 0.1a	81.1 ± 0.2a	13.0 ± 0.4efghi
’15	#1203B21*	*ss2a^L SS3a GBSS1 be2b*	59.2 ± 0.1cd	68.0 ± 0.1de	75.3 ± 0.3d	13.8 ± 0.4cdefg
’15	#1203B23*	*ss2a^L SS3a GBSS1 be2b*	58.8 ± 0.2d	68.3 ± 0.1cd	76.0 ± 0.2cd	13.1 ± 0.4efghi
’15	#1203C11*	*SS2a SS3a GBSS1 be2b*	59.9 ± 0.4c	68.9 ± 0.1b	76.2 ± 0.2c	15.0 ± 0.5bcd
’15	#1203C32*	*SS2a SS3a GBSS1 be2b*	59.7 ± 0.3cd	69.2 ± 0.1b	77.4 ± 0.2b	15.2 ± 0.3abc
’15	#1206A11	*SS2a ss3a gbss1^L be2b*	54.9 ± 0.3hi	64.9 ± 0.1f	71.7 ± 0.4gh	8.4 ± 0.6m
’15	#1206A51	*SS2a ss3a gbss1^L be2b*	52.5 ± 0.3l	63.1 ± 0.3i	71.2 ± 0.2hi	6.7 ± 0.3m
’15	#1206B92	*ss2a^L ss3a GBSS1 be2b*	54.8 ± 0.2hi	63.3 ± 0.1i	70.6 ± 0.1ij	12.5 ± 1.1ghij
’15	#1206B93	*ss2a^L ss3a GBSS1 be2b*	55.7 ± 0.2gh	63.8 ± 0.0h	71.0 ± 0.2hi	10.6 ± 0.3l
’15	#1206C71	*SS2a ss3a GBSS1 be2b*	54.5 ± 0.3ij	64.4 ± 0.0g	73.2 ± 0.2ef	10.5 ± 0.3l
’15	#1206C81	*SS2a ss3a GBSS1 be2b*	56.2 ± 0.5fg	65.1 ± 0.1f	72.4 ± 0.1fg	10.4 ± 0.2l
’15	#1206C83	*SS2a ss3a GBSS1 be2b*	54.8 ± 0.3hi	64.3 ± 0.0g	71.1 ± 0.2hi	10.8 ± 0.1kl

Data represent mean ± standard error (n = 3). Different lowercase letters indicate significant differences among rice genotypes (P <0.05; Tukey-Kramer method). *To*, onset temperature; *Tp*, peak temperature; *Tc*, conclusion temperature; ΔH, gelatinization enthalpy of starch. ’15 and ’16 are harvest years. *Data from Itoh et al. 2017.

Table S6. RS contents of raw and cooked rice flour and un-mashed and mashed cooked rice grains.

Line	Genotype	Raw rice flour	Gelatinized rice flour	Un-mashed cooked rice	Mashed cooked rice
Akita 63	*ss2a*^{L} *SS3a gbss1*^{L} *BE2b*	0.1 ± 0.0f	0.2 ± 0.0f	0.4 ± 0.0c	0.7 ± 0.0g
Kasalath	*SS2a SS3a GBSS1 BE2b*	1.9 ± 0.5ef	1.9 ± 0.0e	2.7 ± 0.1c	2.7 ± 0.1fg
EM10 (BC_3)	*ss2a*^{L} *SS3a gbss1*^{L} *be2b*	15.5 ± 0.5c	1.3 ± 0.1ef	27.8 ± 0.9ab	6.2 ± 0.1de
#4019 (BC_3)	*ss2a*^{L} *ss3a gbss1*^{L} *be2b*	4.7 ± 0.1e	1.4 ± 0.1ef	14.7 ± 0.5bc	3.1 ± 0.1fg
#1203A (BC_3)	*SS2a SS3a gbss1*^{L} *be2b*	21.0 ± 0.3b	1.0 ± 0.1ef	27.8 ± 3.9ab	5.1 ± 0.4ef
#1203B (BC_3)-1	*ss2a*^{L} *SS3a GBSS1 be2b*	28.0 ± 0.4a	9.6 ± 0.4bc	35.2 ± 0.5a	15.4 ± 0.2a
#1203B (BC_3)-2	*ss2a*^{L} *SS3a GBSS1 be2b*	23.5 ± 0.3b	8.3 ± 0.2c	30.7 ± 0.2a	14.7 ± 0.5a
#1203C (BC_3)-1	*SS2a SS3a GBSS1 be2b*	28.0 ± 0.2a	10.4 ± 0.1ab	30.5 ± 2.7a	14.0 ± 0.4a
#1203C (BC_3)-2	*SS2a SS3a GBSS1 be2b*	28.8 ± 0.4a	11.2 ± 0.1a	28.9 ± 1.5ab	12.8 ± 0.4a
#1206A (BC_3)	*SS2a ss3a gbss1*^{L} *be2b*	11.9 ± 0.3cd	4.8 ± 0.0d	27.3 ± 1.2ab	7.3 ± 0.2cde
#1206B (BC_3)	*ss2a*^{L} *ss3a GBSS1 be2b*	10.4 ± 0.2d	6.1 ± 0.1d	29.3 ± 0.1ab	8.0 ± 0.2cd
#1206C (BC_3)	*SS2a ss3a GBSS1 be2b*	30.3 ± 0.2a	6.3 ± 0.2d	32.4 ± 0.2a	9.9 ± 0.3bc

Data represent mean ± SE (n = 3). Different lowercase letters indicate significant differences among rice genotypes ($P < 0.05$; Tukey-Kramer method).
